# Are there non-verbal signals of guilt?

**DOI:** 10.1371/journal.pone.0231756

**Published:** 2020-04-24

**Authors:** Eglantine Julle-Danière, Jamie Whitehouse, Alexander Mielke, Aldert Vrij, Erik Gustafsson, Jérôme Micheletta, Bridget M. Waller

**Affiliations:** Department of Psychology, University of Portsmouth, Portsmouth, United Kingdom; University of Otago, NEW ZEALAND

## Abstract

Guilt is a complex emotion with a potentially important social function of stimulating cooperative behaviours towards and from others, but whether the feeling of guilt is associated with a recognisable pattern of nonverbal behaviour is unknown. We examined the production and perception of guilt in two different studies, with a total of 238 participants with various places of origin. Guilt was induced experimentally, eliciting patterns of movement that were associated with both the participants’ self-reported feelings of guilt and judges’ impressions of their guilt. Guilt was most closely associated with frowning and neck touching. While there were differences between self-reported guilt and perception of guilt the findings suggest that there are consistent patterns that could be considered a non-verbal signal of guilt in humans.

## Introduction

Humans are highly cooperative with both relatives and strangers [1], and the need for cooperation may have provided a powerful selection pressure behind many of the behaviours that we consider uniquely human. Guilt is an emotional and cognitive experience arising when someone feels that they did something wrong. It could have evolved due to its potential adaptive function, within social interaction, of stimulating pro-social behaviours towards and from others. Guilt is classified as a moral, self-conscious emotion, along with pride, shame and embarrassment [2], and is one of the most social, other-oriented emotions that people experience throughout life [3,4]. Despite a growing literature on the social consequences of feeling guilty [4–6], little is known about the behavioural mechanisms underpinning the social functions of guilt, specifically whether guilt is associated with a specific facial expression or nonverbal signal that others can recognise. If people can recognise guilt in others, this might explain how guilt can facilitate cooperation and pro-social behaviours within social interaction. People often state that they can detect a feeling of guilt in others [7], but with some notable exceptions [8], to date, a facial expression of guilt has not been identified scientifically.

Moral emotions are thought to facilitate the complex navigation of social interactions and relationships [9–11], allowing one to consider behaviour in light of social norms and the differing perspective of others. Early accounts of guilt cast it primarily as a self-regulatory emotion prompting individuals to reflect on their mistakes and ultimately feel better about themselves [4]. It has been shown to have a potentially positive function within social interaction of stimulating pro-social behaviours towards and from others, promoting actions towards those who have been wronged specifically [4–6]. Experiments have shown that guilt can prompt people to specific actions towards others, such as helping behaviours [12] and prejudice-reducing behaviours [13]. The virtue of apologies and verbal admittance of wrong-doing has been well studied [14,15], revealing that people (victims of wrong-doing or by-standers) would be more lenient towards a wrong-doer that recognise their faults. For example, in legal contexts, judges and jurors claim that they know when a defendant is sorry for the crime they have committed [7], which can then impact on sentencing. Guilt, however, is not just a social emotion. Indeed, a *Dobby Effect* has been highlighted [16], refuting the all-social aspect of guilt, and showing that guilty people sometimes punish themselves in the absence of opportunity to make amends to the victim of their wrong-doing. The social aspect of guilt seems then linked to the context the guilty person finds themselves in: they will act pro-socially and make amends in social contexts but will engage in self-punishment when socially isolated [16]. Finally, guilt can be experienced automatically after committing a social transgression (self-induced), but can also be induced by others as a method of control to gain power within relationships [other induced; 17]. Guilt can thus be a complex and powerful phenomenon within social negotiations, but whether guilt can be observed by others without being explicitly declared is unknown. If guilt can be detected in this way, the potential to affect social outcomes between individuals is increased.

Whether emotions (and which emotions) are associated with universally produced and recognised facial expressions is debated. The classic and largely dominant view, the Basic Emotion Theory [BET; 18,19,20], is that primary, basic emotions [happiness, sadness, anger, surprise, disgust and fear - 19] are considered innate to all human populations and universally expressed [20,21], and so likely resulting from specific functional adaptations [22]. In contrast, secondary emotions (of which guilt is one, along with embarrassment, shame, and contempt) are thought to differ significantly between cultures [23,24], their expressions subject to specific cultural display rules [20,24], and acquired and developed gradually during childhood [25]. Secondary emotions are more idiosyncratic and context-dependent, which is why it has been difficult to identify specific facial movements associated with the experience of those emotional states. The later ontogeny led scientists to explore the possible influence of environment on the development of secondary emotions [20], and through the impact of these variable environments, they are not thought to have a prototypical universal expression [26]. Within a Behavioural Ecological View of facial expressions [BEV; 27,28], however, the distinction between primary and secondary emotions is less rigid. BEV argues that facial expressions indicate the sender’s most likely future behaviours (i.e., action tendencies) and thus function as important social signals in social interaction. Facial expressions benefit both the sender and receiver by reducing the need for conflict when interests are declared openly [1,17,27–30]. As such, both primary and secondary emotions *can* be associated with specific, readable, and recognisable facial signals, as it is not the emotion per se that is being transmitted, but instead the potential social action [28,30]. Therefore, if guilt is associated with a specific social outcome (e.g. making amends, increased likelihood to cooperate in the future), people could detect this from nonverbal behaviour, specifically from a facial signal with communicative value. Signals can therefore be understood as a way for an individual to manipulate or alter the behaviour of another individual [31–33]. Signals can also be used by others when deciding if and how to respond to a given situation [34]. The potentially important role of the face in social interactions led us to hypothesise that guilt would be associated with an identifiable facial signal (i.e., facial expression), and that non-verbal signals (i.e., self-directed behaviours) could also be present.

A non-verbal signal can include not only facial expressions (i.e., resulting from the contraction of specific facial muscles), but also head position, behaviours directed towards the head (e.g., touching the face or hair), body postures and gestures. Non-verbal behaviours (focussing here on facial expressions and actions directed towards the face) can be considered a signal if those behaviours are reliably associated with the experience of guilt and are accurately perceived by observers as an indication of guilt, as well as influencing the observers’ behaviours [33]. Here, we tried to identify non-verbal signals resulting from a specific cognitive appraisal (i.e., a situation designed to induce guilt; [35]), occurring concomitantly with a self-reported feeling of guilt. By doing this, we are following Scherer et al. [35]’s view that non-verbal signals can carry emotional meaning, as well as action tendencies which can both be perceived and interpreted by observers. Moreover, some researchers argue that the concept of emotion is constructed [36–38] as the result of a given experience, at a specific time, in a specific context [37]. As such, both theories [35,37] advocate for a less direct link between non-verbal signals and emotional states than previously argued by the Basic Emotion Theory [19], while still expecting non-verbal signals to have potential function and meaning.

Some secondary emotions [e.g. shame and embarrassment; 8,39] have been associated with recognisable facial movements, but these emotions are often confused with each other. Guilt can also be mistaken or mislabelled as shame, and sometimes embarrassment, and research has tried to differentiate between those, not only in terms of the psychological meaning but also in terms of the behavioural signal [8,25,40]. The specific social context in which the facial expression is placed therefore can be important in the interpretation of these expressions [41,42]. Nevertheless, there must be some key physical elements to such expressions that underpin their recognition to make them in some way identifiable to others.

For instance, the action tendencies of shame, embarrassment and guilt, are rather different, and may thus manifest as physical differences in a behavioural signal. Behavioural responses to embarrassment and shame have been identified over the years [43–45]: embarrassment displays are marked by gaze down, controlled smiles, gaze shifts, and face touches [44], whereas a shameful display is marked with head and gaze down [43–45]. Embarrassment serves a reconciliatory and appeasement function, reconciling in social relations following transgressions [see 46 for review], whereas shame serves a reconciliatory and appeasement function following hierarchical transgressions. In contrast, a facial expression of guilt has not been clearly described. Guilt may have evolved in humans due to the value in indicating one’s willingness to make amends. Only one study has tried to identify a recognisable set of facial movements associated with the experience of guilt [8]. Three potential displays of guilt were presented on still photographs: a facial expression representing self-contempt, which has been shown to be associated with the experience of guilt [47]; a non-verbal display of sympathy [48], which could be part of the experience of guilt; and finally a facial expression of pain, considered as one antecedent of guilt [49]. Following the presentation of the still photographs, participants in this study had to select one emotion word among 14 different options (including a “no emotion” option). None of these conceptualised displays of guilt were identified as such by observers [8]. The authors speculated that participants may have struggled with identifying fixed displays compared to spontaneous dynamic stimuli of the same emotions [8,44]. This study motivated us to try a new methodology, with a bottom-up approach to try inducing guilt in the laboratory to collect spontaneous dynamic displays associated with the experience of guilt that we could then present to naïve observers.

### Present investigation

Here, we examined variation in both the production and perception of the specific facial movements associated with guilt in a culturally diverse sample including participants with different geographic backgrounds, recruiting people from WEIRD and non-WEIRD countries [Western, Educated, Industrialised, Rich and Democratic societies; 50]. We examined the production and perception of spontaneous facial expressions using a bottom-up approach to identify dynamic patterns in facial behaviour, departing from the classic method of coding the apex of an expression or movements of interest only [51,52]. We looked at the production of facial movements in individuals currently living in the UK but belonging to different cultures and originating from different countries to assess overall patterns produced in association with guilt and gain general knowledge, regardless of the origin of individuals. Firstly, we identified facial movements based on what people displayed when experiencing guilt. Secondly, we identified facial movements based on what people perceived as guilt. This study looked at the production of a facial expression of guilt using for the first time an experimental induction approach and an extensive dynamic facial movement coding system.

## Study 1 –production of guilt

### Methods

#### Participants

One hundred and thirty-one participants took part in this study (94 females; *M*_*age*_ = 25.41, *SD* = 9.47; see S1 Study *of Table 1* in *Supplementary Materials* for details). Participants were recruited based on an opportunistic sampling method and were all UK resident at the time of the experiment (but included both UK and non-UK nationals). All of them received either course credit (if student) or £5 for their time. The whole experiment lasted 45 minutes on average. Participants had various ethnicities and nationalities, constituting a sample made of individuals with various Places of Origin [PoO—see S1 Study of Table 1 for details; 50]. The project has been reviewed and approved by the Science Faculty Ethics Committee (SFEC) from the University of Portsmouth. Each participant signed an informed consent form granting authorisation for the use of the data for research purposes. The individuals pictured in in this manuscript (Fig 2 and S1 Video) have provided written informed consent (as outlined in PLOS consent form) to publish their image alongside the manuscript.

#### General procedure

**Fig 1:**
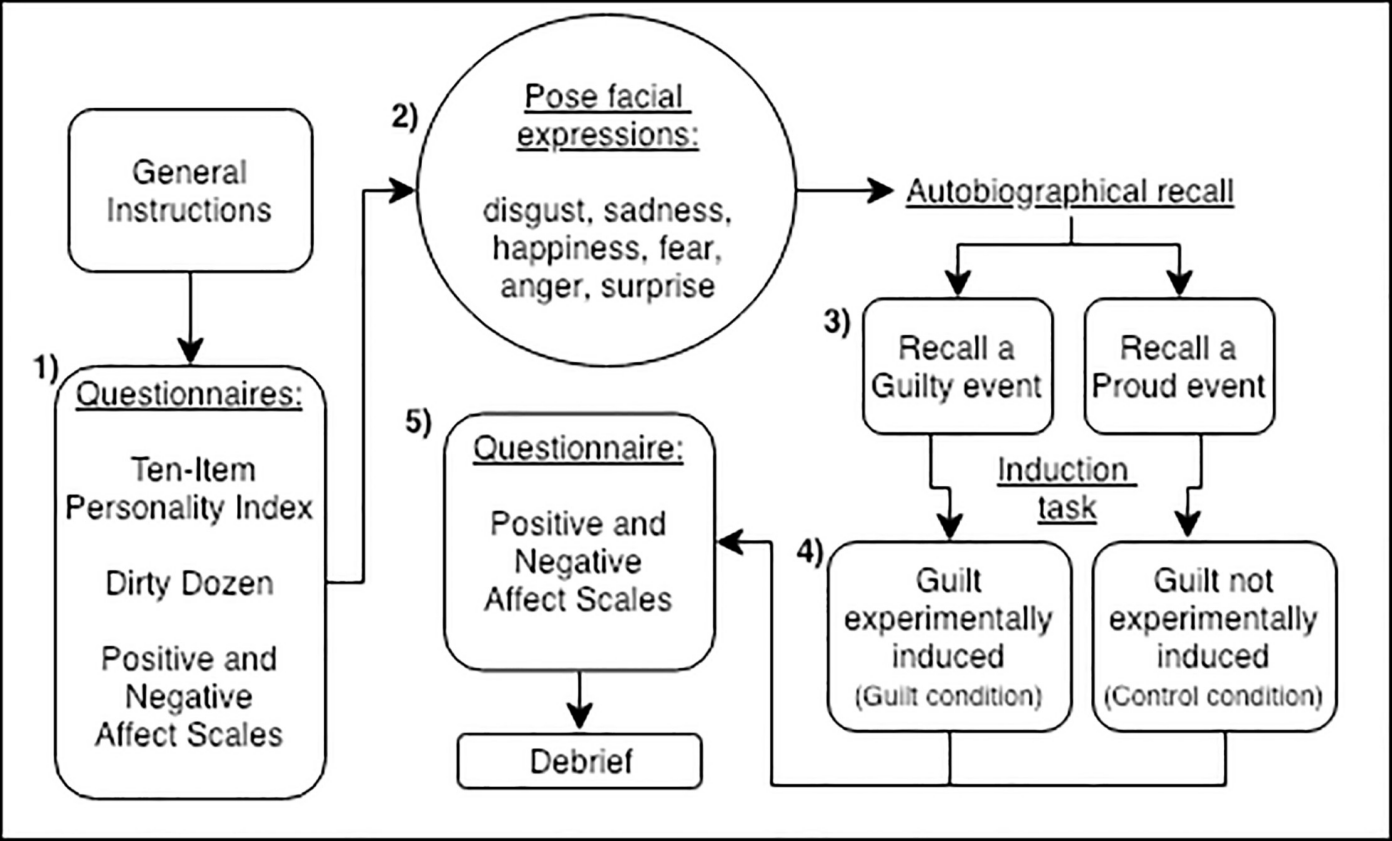
General procedure. A flowchart representing the procedure of the experiment in Study 1.

To begin, participants were given general instructions regarding the experiment and written consent was obtained. Participants were originally told that this study had a different aim—to assess how personality affects behaviour and facial expressions. Following these instructions, the rest of the tasks were displayed on a computer using the OpenSesame© software [53], and the participant was filmed for the remaining time (using a JVC Everio GZ-MG750, 25 frames/second, placed approximately 50 cm away from their face). The experiment consisted of 5 key steps, as outlined in Fig 1 and explained in more detail below. Participants were fully debriefed at the end of the experiment.

First (Fig 1: 1), participants were required to complete two personality questionnaires, the Ten-Item Personality Index [TIPI; 54] and the Dirty Dozen [DD; 55], followed by a mood-check questionnaire [Positive and Negative Affect Scales—PANAS; 56]. Question order was randomised between individuals. Personality questionnaires were used as part of our cover-up story (examining the impact of personality on behaviours and facial expressions); to investigate whether main personality traits correlated with self-reported guilt (see Supplementary Material for the results). Second (Fig 1: 2), participants were prompted to pose six emotional facial expressions (anger, fear, disgust, happiness, surprise, and sadness) in succession and hold each of them for 8 seconds. This acted as a filler task to disassociate the participants from the previous questionnaires. We used a video camera to record facial expressions of emotional states produced in this task. The experimenter then pretended to turn off the camera, but in reality, kept recording the rest of the experiment. Third (Fig 1: 3), participants were asked to recall an autobiographical event, and write about this in some detail. Participants were prompted to either recall an event where they felt guilty regarding something they did for a relative (guilt condition), or an event where they felt proud (control condition). Participants were randomly assigned to a single condition (guilt or control). This third task was used as both a priming task to start inducing either guilt or pride in participants [as used in previous research; 57], and was a necessary component of the following induction task. Fourth (Fig 1: 4), in participants who were assigned to the guilt condition, and recalled a guilty autobiographical event, guilt was induced further experimentally. Here, the experimenter asked the participant to save their written recall on a USB flash drive. Once returned to the experimenter, the participant was informed that the USB flash drive had become corrupt, and their data, among all the other data of other participants in the study, had been lost. During this social interaction between the experimenter and participant, it was clearly implied that it was the participant’s fault. They were told that this would be reported to the Principal Investigator and that there was nothing to be done at present. They were finally invited to resume the experiment. In the control condition, guilt was not induced, and participants were told that their written recall was saved correctly on the USB flash-drive and were asked to continue. Finally, (Fig 1: 5), participants completed a second PANAS questionnaire, after which they were informed about the true aim of the experiment, told that the camera had kept on recording and debriefed.

As mentioned, previous research used autobiographical recall as an induction task, relying on the fact that remembering a previous emotional state can elicit said emotion again [57]. Using this methodology, group differences have been found between guilt-recall and control-recall [6,58]. However, inducing an emotional state in the present is more ecologically valid and might standardise the feelings of guilt across participants to a greater extent [57]. Building on previous research [5], we therefore decided to use the autobiographical recall as a priming task, to get participants to start thinking about one of two emotions (pride or guilt), and then the experimental induction as a standardised induction of state guilt (i.e., feelings of guilt). We chose a positive secondary emotion for the control condition to make participants think about the recalled event in both conditions. We chose pride for the control condition as both pride and guilt are categorised as secondary emotions [2], presenting similar levels of arousal but opposite valence [pride being positive whereas guilt is negative; 2]. Asking participants to recall an event of their day (e.g., their breakfast) did not seem as strenuous or emotional as recalling a time they felt guilt for something they did. The analysis of the autobiographical recalls is not presented here, but the length of the recalls was similar in the guilt (*M* = 147.4 words; SD = 99.36) and pride (*M* = 136.5 words; SD = 79.54) conditions (*p* = 0.5). This made us confident that the involvement in writing the recalls was similar between the conditions.

#### Coding of facial movements

Videos collected during the experiment were first cropped to extract the relevant moment only: the induction task (Fig 1: 4); and were coded for facial movements using the Facial Action Coding System [FACS; 59,60]. Self-directed behaviours (face and neck touch) were also coded due to their direct links to negative affective states [61]. All facial movements produced by the participant during the induction task (Fig 1: 4) were coded for Action Units (single muscle movements; AUs) or Action Descriptors (one or more unspecified muscle movements; ADs), in both the guilt (average duration = 73.66 sec; *SD* = 46.56 sec; see Video1) and the control (average duration = 7.90 sec; *SD* = 4.27 sec) conditions. The bootstrapping approach chosen for statistical analyses (detailed below) accounts for the fact that the responses in the two conditions differ in duration. Videos of posed emotional facial expressions were FACS coded and used for the reliability, but were not analysed further. A full list of coded movements, defined by the FACS, can be found in S1 Study of Table 2. A heat map presenting AUs produced through time is presented in S1 Fig. Blushing was originally part of the ethogram but as no occurrence was observed, it was removed from further analysis. Coding was conducted on each frame of the videos by the first author. During coding, the first author was blind to the conditions.

For every participant, we obtained the total number of frames of different AU/ADs produced (i.e., the number of instances, from start to end, for each given AU/AD) in a given condition as well as the overall duration (i.e., the total time the AU/AD was expressed on a face) an AU/AD was produced for [62]. We were also able to extract temporal data, giving us the state of each AU/AD at a given frame in the video (absent, present at small intensity or present at high intensity; 25 frames per second). All coding was conducted using the Interact© software [63].

We used a binomial exact test as criteria for exclusion of specific facial muscles from subsequent analyses—if any AU/AD was produced by fewer participants than the calculated criteria (here, the criteria given by the binomial test was a minimum of 39 participants in both conditions), this AU/AD was not explored further in an attempt to maintain a robust dataset. The binomial exact test allowed us to keep facial movements produced significantly more than chance: if at least 39 participants produced the movement, then this movement reliably occurs across participants and did not result from individual differences. Based on previous literature associating the experience of guilt with the experience of self-contempt, and pain [8] and the common confusion between shame and guilt [8,25,40], we also kept AUs previously shown to be associated with shame [AUs 54+64; 8] and self-directed behaviours, previously associated with stress/pain and discomfort (neck and face touching). After the binomial test, 24AU/ADs out of a possible 39 observed in our data (see S1 Study of Table 2 and S1 Fig) and 117,781 frames were left in the guilt condition (12,472 frames in the control condition) for further analysis.

We conducted inter-rater reliability testing between the main coder (first author) and a second coder, both of which are trained FACS coders). Reliability analysis on these 15 AUs was conducted on 5% of the video clips extracted from the videos collected during the study (42 of 820 videos, half of which were from the posed facial expression task and half from spontaneous facial expressions during the induction task, from both control and guilt conditions). Reliability analysis is important for FACS coding to ensure that the coding is unbiased, and all the produced movements were observed and reported by the main coder. For analysis, we calculated the Krippendorff’s alpha [64,65] using the “KAlpha” macro for use with IBM SPSS version 24 [66]. Krippendorff's alpha coefficients are considered reliable if the 95% confidence was greater than chance (i.e., if the lower bound was >0). According to this index, the reliability coefficient was significantly greater than chance (α = 0.740; K-α 95% LCI: 0.684; K-α 95% UCI: 0.788), indicating that the two FACS coders shared a good reliability in their coding judgements given the coding scheme used here (full FACS coding, with duration and intensity). This K’s alpha is higher than the lowest acceptable limit (α ≥ 0.667) but is under the customary required benchmark (α ≥ 0.800); our results should thus be interpreted with caution and provide preliminary results regarding facial movements associated with the experience of guilt [67,68].

#### Statistical analysis

*Guilt induction*. To test for the success of the induction of guilt during the guilt induction task, we compared the affect data collected through the PANAS questionnaires (before vs. after induction) using a within-subjects *t*-test. We tested for a change in positive and negative affect before vs. after induction, and additionally, some specific emotional changes in guilt, shame, distress, and pride, which were all measured in the PANAS questionnaire.

*Facial expressions*. The likelihood of an action unit to be active during any communication event is likely influenced by a several interdependent factors. Among those, we can find the information that is transmitted and the context; inter-individual and cultural differences; temporal effects; the intensity of stimuli; random variation in expression; duration of expressions; interdependence in the co-occurrence of action units; and anatomical limits in which action units can be used at the same time. Furthermore, the likelihood of occurrence of any action unit is interdependent from the likelihood of using a certain number of action units at the same time. Statistical approaches that are often used when analysing FACS data make assumptions about the distribution of the underlying data (continuous variables, independence of cases) that are rarely met in facial expression datasets. Rather than testing whether the distributions of action units differ in samples within the confines of existing variance tests, researchers can use permutation and bootstrapping procedures that allow for controlling some of the aforementioned factors and provide statistically accurate measures of significance [69]. Here, we employ a bootstrapping approach to test whether action units differ between the experimental conditions (guilt or control) of this study.

The FACS coding information of the participant videos was structured frame-by-frame (as it was coded), with each selected action unit representing one column and their presence or absence coded as 1 or 0, respectively. Retaining the frame information means that facial expressions that are shown for longer influence the results accordingly. Frames in which it was not possible to see the whole face were removed. Frames presented between 0 and 15 of the selected action units (see AU selection above) active at the same time. All statistical tests here are direct comparisons of two distributions, to see whether they stem from the same or different underlying populations: a control distribution (e.g., control condition) and a test distribution (e.g., guilt condition), with the question invariably being whether the frequency of occurrence of any given action unit differs between the former and latter.

We applied a bootstrapping procedure to create the probability distribution of the occurrence of each action unit under the null hypothesis that they are from the same distribution as the control condition: by repeatedly taking random subsets of the control data, we establish a range of values the frequency of occurrence of an action unit could take if it was drawn from this population. We randomly selected individuals in the control condition [sampling with replacement approach; 69] to account for the fact that there might be inter-individual differences in expressivity or use of action units. Thus, each individual in the control condition was sometimes included and sometimes excluded in generating the control distribution, ascertaining that the distribution was not skewed due to the properties of certain individuals. Cultural differences in the use of facial expressions [70–72] and the self-report of emotions [73,74] might exist and data might thus be following a hidden structure due to participants' PoO. Participants were clustered into two regions for PoO: European and East Asian. We balanced the assignment of individuals to the control distribution. We established the ratio of PoOs of participants in the test dataset and applied the same ratio to the control distribution. Thus, if for example the test dataset included ten participants who reported East Asian origins and five participants of European origins, then each randomised control dataset would maintain the 2-to-1 ratio between the two groups.

Using this procedure, we created 1000 bootstrapped control distributions for each statistical test that have the appropriate underlying data structure and address potential problems arising from inter-individual and cultural differences. We established the frequency of occurrence for each action unit for the test data (observed frequency) and for each action unit over all 1000 bootstraps (expected frequency if the data would arise from the same population). To test whether the frequency of occurrence was significantly higher or lower than expected, we report the z-value of the observed frequency compared to the control distribution (i.e., how many standard deviations does it differ from the mean). We assumed that the null hypothesis (the observed value for the test data is part of the same distribution that created the control condition) was rejected if the observed value was more extreme than 99% of bootstrapped values (two-sided testing). The p-value represents the likelihood of the observed frequency of an action unit in the test condition being lower or higher than the expected frequency of each bootstrap. We set our significance level at 0.01 to account for multiple testing while avoiding false rejections [75]. A p-value of 0.01 and a positive z-value indicates that in 990 out of 1000 bootstrapped selections of the control data, the action unit occurred less frequently than in the test data.

We tested four questions using this approach: first, we tested the overall difference between the control condition and the guilt condition of the experiment, to see whether there were differences in the facial expressions between experimental interventions. However, there were considerable differences between individuals in their reported feeling of guilt before and after the intervention in the guilt condition, with some individuals not reporting an increase in guilt (*self-reported guilt after induction—self-reported guilt before induction ≤ 0*). Thus, secondly, we investigated individuals who did not show any change in reported guilt (‘weak guilt’ sample, N = 19) and individuals who showed an increase in reported guilt (‘strong guilt’ sample, N = 45) separately to test whether these differences in reported guilt also showed in the facial activity. We tested both datasets against the control dataset (to establish whether guilt induction worked in the former group), and we finally tested the two guilt samples against each other to see if stronger reported guilt led to increased production of some action units. As with the bootstrapping approach we are testing whether the distribution of each AU in the weak guilt sample and the strong guilt sample could stem from the control group, having more participants reporting an increased feeling of guilt will not impact the analysis conducted.

### Results

#### Guilt induction

In our guilt condition, participants reported more negative affect after (*M* = 21.89, *SD* = 8.23) the guilt induction task (Fig 1.1:4) compared to before (*M* = 18.61, *SD* = 8.56; t(65) = -2.68, p < 0.001). They also experienced a decrease in positive affect after the induction (*M* = 20.27, *SD* = 8.08) compared to before (*M* = 29.73, *SD* = 6.12; t(65) = 9.02, p < 0.001).

More specifically, we found an increase in guilty feelings after the induction task (*M* = 2.7, *SD* = 1.23) compared to before (*M* = 1.35, *SD* = 0.79; t() = -8.31, p < 0.001; see S1 Study of Table 3). Participants also reported higher levels of shame after the induction (*M* = 2.24, *SD* = 1.12) compared to before the interaction with the experimenter (*M* = 1.33, *SD* = 0.83; t(65) = -5.91, p < 0.001). Participants reported a significantly higher level of guilt than shame after the induction task (t(65) = -3.00, p = 0.0038).

Finally, participants reported an increase in distress after (*M* = 2.42, *SD* = 1.15) the induction task compared to before (*M* = 1.58, *SD* = 0.95; t(65) = -5.29, p < 0.001), as well as a decreased pride after (*M* = 1.89, *SD* = 1.89) the induction compared to before (*M* = 2.35, *SD* = 1.06; t(65) = 3.84, p < 0.001).

In the control condition, participants reported less positive affect after the interaction with the researcher (*M* = 25.18, *SD* = 11.06) compared to before (*M* = 30.46, *SD* = 8.47; t(64) = 4.11, p < 0.001), but they also experienced a decrease in negative affect after the induction (*M* = 12.88, *SD* = 4.97) compared to before (*M* = 21.2, *SD* = 10.96; t(64) = 6.44, p < 0.001; see S1 Study of Table 3).

More specifically, we found an decrease in distress after (*M* = 1.48, *SD* = 0.89) the induction task compared to before (*M* = 1.72, *SD* = 0.98; t(64) = 2.34, p = 0.0225), as well as increased pride after (*M* = 3.4, *SD* = 1.2) the induction compared to before (*M* = 2.46, *SD* = 1.15; t(64) = -6.78, p < 0.001).

When comparing the affect data collected after induction between the control and the guilt conditions, participants reported higher positive affect (*M* = 4.82, *SE* = 1.65; t(65) = 2.93, p = 0.004) and pride (*M* = 1.26, *SE* = 0.20; t(65) = 6.28, p < 0.001) in the control condition. Moreover, they reported lower negative affect (*M* = -5.72, *SE* = 1.33; t(65) = -4.30, p < 0.001) in the control condition than in the guilt condition.

More specifically, they reported lower guilt (*M* = -1.42, *SE* = 0.174; t(65) = -8.34, p < 0.001), distress (*M* = -0.52, *SE* = 0.19; t(65) = -2.70, p < 0.001), shame (*M* = -0.37, *SE* = 0.18; t(65) = -2.17, p < 0.001), and nervousness (*M* = -0.63, *SE* = 0.17; t(65) = -3.62, p < 0.001) in the control condition compared to the guilt condition [*means and SE presented characterise the difference between the values in the control and the values in the guilt conditions*]. These results confirmed the effectiveness of the guilt induction method used; participants exposed to the guilt induction task reported higher levels of guilt and associated negative affect compared to those that were in the control condition.

#### Comparison of guilt and control conditions

**Fig 2:**
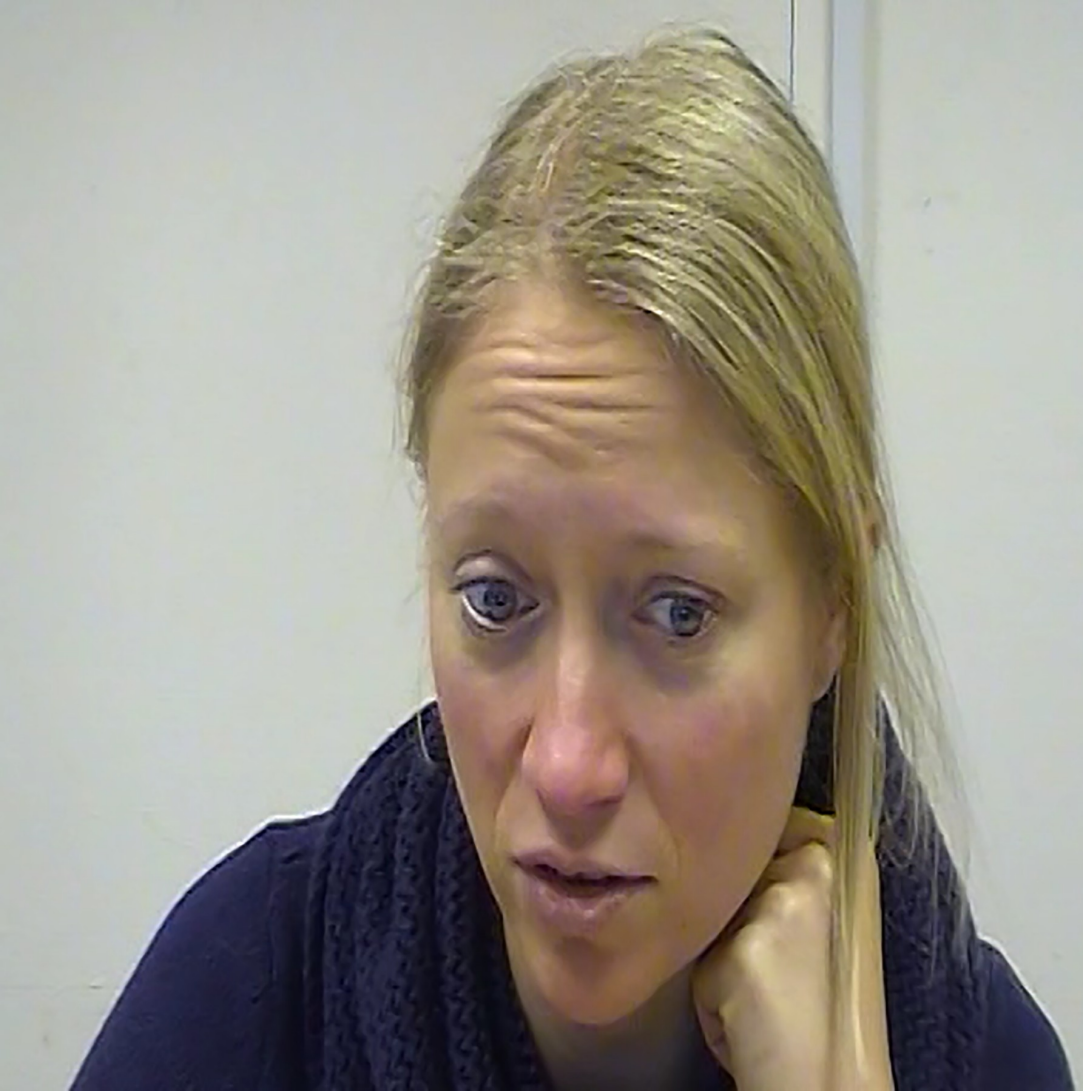
Images of guilty expressions taken from a video. AUs 1+4+10+12+(20+)25+26+Neck Touch produced in this image; the perceived production of AU20 might be due to speech at the same time (participant apologising). *The individual pictured in Fig 2 has provided written informed consent (as outlined in PLOS consent form) to publish their image alongside the manuscript*.

The results of the bootstrap test, creating expected distributions for action units based on the control condition and comparing those with the observed distribution of action units in the guilt condition, revealed that participants in the guilt condition exhibited facial muscle activation that was significantly different from the control condition. Table 1 presents the summary of the comparison for the entire guilt dataset. In the upper face, AU4 (Brow Lowerer) was more active in the guilt condition, produced more than twice as often as in the control condition. In the lower face, AU20 (Lip Stretch) was active more often than would have been predicted based on the control condition. Participants in the guilt condition turned their eyes and heads to the right (AU52 –Head Turn Right, AU62 –Eyes Turn Right) more than predicted. Most striking was the difference in the likelihood of participants to touch their neck, being almost twenty times more likely in the guilt condition than expected (see Fig 2). There was a trend for participants to touch their face more than expected. Participants in the guilt condition were significantly less likely to show activation of AU12 (Lip Corner Puller), AU14 (Dimpler), AU17 (Chin Raiser), AU51 (Head Turn Left), AU57 (Head Forward), AU61 (Eyes Turn Left), and AU64 (Eyes Down). Thus, those movements (presented in italics in Table 1) were consistently more produced in the control condition and are not specific to the experience of guilt.

**Table 1:** Comparison of the full dataset for the guilt condition (observed frequency) with the predicted distribution based on the control condition (expected frequency), after controlling for differences in the PoOs of participants.

AU	Observed Frequency	Expected Frequency	z	p-value
1	0.52	0.51	0.33	0.357
2	0.50	0.50	0.18	0.418
**4**	**0.19**	**0.08**	**8.82**	**0.000**
5	0.12	0.11	0.31	0.381
7	0.26	0.32	-1.48	0.071
10	0.26	0.23	1.12	0.114
*12*	*0*.*14*	*0*.*32*	*-5*.*78*	*0*.*000*
*14*	*0*.*15*	*0*.*24*	*-3*.*66*	*0*.*000*
*17*	*0*.*14*	*0*.*21*	*-3*.*05*	*0*.*003*
18	0.13	0.13	0.03	0.493
**20**	**0.03**	**0.02**	**2.37**	**0.000**
24	0.15	0.13	0.78	0.234
*51*	*0*.*09*	*0*.*29*	*-5*.*66*	*0*.*000*
**52**	**0.29**	**0.22**	**3.22**	**0.000**
54	0.15	0.18	-1.42	0.072
55	0.19	0.23	-1.23	0.112
56	0.17	0.21	-1.09	0.132
*57*	*0*.*16*	*0*.*25*	*-3*.*45*	*0*.*001*
59	0.03	0.03	-1.11	0.135
*61*	*0*.*09*	*0*.*15*	*-4*.*97*	*0*.*000*
**62**	**0.21**	**0.15**	**4.20**	**0.000**
*64*	*0*.*30*	*0*.*39*	*-3*.*58*	*0*.*000*
FaceTouch	0.13	0.10	1.60	0.041
**NeckTouch**	**0.02**	**0.00**	**25.69**	**0.000**

P-values denote the likelihood that the observed frequency of occurrence for an AU was more extreme than the predicted frequency. AU with significantly increased occurrence in bold, AU with significantly reduced occurrence in italics.

We found no significant relationship between self-reported shame or pride and AU4 (shame: ß = 3.36e-4; SE = 3.81e-4; p = 0.381; pride: ß = 3.46e-5; SE = 3.28e-4; p = 0.916), AU20 (shame: ß = -8.37e-4; SE = 1.56e-3; p = 0.593; pride: ß = 1.20e-3; SE = 1.34e-3; p = 0.376), AU52 (shame: ß = -4.11e-4;SE = 2014e-4; p = 0.210; pride: ß = 1.88e-4; SE = 2.78e-4; p = 0.502), AU62 (shame: ß = 1.04e-3; SE = 6.36e-4; p = 0.109; pride: ß = -2.58e-4; SE = 5.47e-4; p = 0.639), or Neck Touch (shame: ß = 1.37e-3; SE = 1.45e-3; p = 0.347; pride: ß = -1.37e-3; SE = 1.24e-3; p = 0.273). Thus, when participants reported high level of shame (or pride), the facial movements produced were not the ones they produced when reporting high level of guilt, confirming that the movements identified here are more specific to the experience of guilt that to the experience of shame.

#### Comparison of weak guilt and strong guilt samples

Participants reported different levels in how much their feeling of guilt changed due to the experimental intervention, with several participants not reporting any increase in feelings of guilt. Thus, we tested whether participants who reported no increase in feeling guilty still differed significantly from the control condition, whether the observed changes were equivalent to those in participants who reported increased feelings of guilt, and how the two subgroups differed from each other.

As reported in Table 2, while there were some differences between the participants who reported guilt and those who did not, both groups showed increased activation in AU4, AU20, AU62, and both touched their necks more than expected given the control condition. Face touching only occurred significantly more than expected in the weak guilt condition, while AU52 occurred more frequently in individuals who reported an increase in guilt after induction.

**Table 2:** Comparison of the dataset for participants who reported no change in feelings of guilt (‘weak guilt’) and the dataset for participants who reported changes in feelings of guilt (‘strong guilt’) with the predicted distribution based on the control condition (expected frequency) after controlling for differences in the PoOs of participants.

Weak Guilt					Strong Guilt			
AU	Observed Frequency	Expected Frequency	z	p-value	Observed Frequency	Expected Frequency	z	p-value
1	0.55	0.52	0.84	0.199	0.51	0.51	-0.04	0.472
2	0.57	0.51	1.43	0.090	0.48	0.50	-0.52	0.297
**4**	**0.29**	**0.08**	**10.56**	**0.000**	**0.17**	**0.08**	**5.77**	**0.000**
*5*	*0*.*07*	*0*.*12*	*-2*.*32*	*0*.*009*	0.15	0.11	1.53	0.064
*7*	*0*.*21*	*0*.*32*	*-2*.*42*	*0*.*008*	0.29	0.31	-0.51	0.308
**10**	0.20	0.23	-0.94	0.177	**0.29**	**0.23**	**1.80**	**0.027**
*12*	*0*.*14*	*0*.*31*	*-4*.*96*	*0*.*000*	*0*.*15*	*0*.*32*	*-4*.*59*	*0*.*000*
*14*	*0*.*16*	*0*.*24*	*-3*.*00*	*0*.*001*	*0*.*16*	*0*.*24*	*-2*.*83*	*0*.*002*
*17*	*0*.*12*	*0*.*21*	*-3*.*56*	*0*.*000*	*0*.*15*	*0*.*21*	*-2*.*10*	*0*.*016*
18	0.15	0.13	0.51	0.307	0.13	0.13	-0.15	0.442
**20**	**0.04**	**0.02**	**2.38**	**0.000**	**0.03**	**0.02**	**1.76**	**0.008**
24	0.13	0.14	-0.55	0.290	0.16	0.14	1.15	0.128
*51*	*0*.*11*	*0*.*31*	*-5*.*22*	*0*.*000*	*0*.*09*	*0*.*29*	*-4*.*68*	*0*.*000*
**52**	0.25	0.21	1.49	0.057	**0.32**	**0.22**	**3.16**	**0.000**
54	0.17	0.18	-0.30	0.387	0.15	0.19	-1.60	0.054
55	0.20	0.23	-0.88	0.188	0.19	0.23	-1.05	0.151
56	0.20	0.21	-0.23	0.412	0.16	0.21	-1.29	0.094
*57*	*0*.*13*	*0*.*27*	*-4*.*59*	*0*.*000*	*0*.*19*	*0*.*25*	*-2*.*12*	*0*.*016*
*59*	*0*.*02*	*0*.*04*	*-2*.*88*	*0*.*002*	0.04	0.04	0.08	0.493
*61*	*0*.*06*	*0*.*15*	*-6*.*37*	*0*.*000*	*0*.*11*	*0*.*15*	*-2*.*87*	*0*.*003*
**62**	**0.24**	**0.15**	**5.31**	**0.000**	**0.20**	**0.15**	**2.52**	**0.003**
*64*	*0*.*30*	*0*.*37*	*-2*.*32*	*0*.*015*	*0*.*30*	*0*.*39*	*-2*.*95*	*0*.*001*
**FaceTouch**	**0.16**	**0.11**	**2.25**	**0.009**	0.12	0.10	0.86	0.214
**NeckTouch**	**0.01**	**0.00**	**10.15**	**0.000**	**0.02**	**0.00**	**26.69**	**0.000**

P-values denote the likelihood that the observed frequency of occurrence for an AU was more extreme than the predicted frequency. AU with significantly increased occurrence in bold, AU with reduced occurrence in italics.

In the direct comparison between the two groups (individuals who expressed changes in feeling of guilt and those who did not; Table 3), individuals who reported strong feelings of guilt were more likely than expected to show activation of AU5 (Upper Lid Raise), AU10 (Upper Lip Raiser), AU 57, AU59 (up-down head shake—nodding) and AU61 (corroborating the results of comparing each of the two with the control condition–Table 2). AU5 was reduced in participants who did report weak guilt compared to control (Table 2). Activation of AU61 was reduced in both subgroups of the guilt condition (compared to control; Table 3) but was significantly more likely to occur in participants who reported strong guilt. AU57 and AU59 also occurred more in the strong guilt condition (Table 3). No action unit occurred significantly more in the weak guilt than in the strong guilt condition: the action units identified were thus associated with feelings of self-reported guilt.

**Table 3:** Comparison of the dataset for participant who reported changes in feelings of guilt (observed frequency) with the predicted distribution based on participants who reported no changes in feelings of guilt (expected frequency) after controlling for differences in the PoOs of participants.

AU	Observed Frequency	Expected Frequency	z	p-value
1	0.51	0.54	-0.58	0.261
2	0.48	0.56	-1.54	0.071
4	0.17	0.24	-1.56	0.058
**5**	**0.15**	**0.08**	**4.34**	**0.001**
7	0.29	0.21	1.25	0.103
**10**	**0.29**	**0.19**	**2.63**	**0.000**
12	0.15	0.15	0.03	0.490
14	0.16	0.15	0.13	0.467
17	0.15	0.14	0.49	0.282
18	0.13	0.15	-0.73	0.228
20	0.03	0.04	-0.25	0.380
24	0.16	0.13	0.95	0.181
51	0.09	0.10	-0.30	0.393
52	0.32	0.25	1.50	0.066
54	0.15	0.17	-0.74	0.255
55	0.19	0.20	-0.17	0.434
56	0.16	0.20	-0.51	0.402
**57**	**0.19**	**0.13**	**2.57**	**0.002**
**59**	**0.04**	**0.02**	**2.39**	**0.004**
**61**	**0.11**	**0.07**	**2.18**	**0.009**
62	0.20	0.24	-1.36	0.079
64	0.30	0.30	-0.17	0.392
FaceTouch	0.12	0.16	-0.75	0.214
NeckTouch	0.02	0.01	1.47	0.065

P-values denote the likelihood that the observed frequency of occurrence for an AU was more extreme than the predicted frequency. AU with significantly increased occurrence in bold, AU with significantly decreased occurrence in italics.

Thus, in summary, there is strong evidence that AU4, AU20, AU52, and AU62, as well as the touching of the neck, were significantly produced more frequently in all participants in the guilt condition (Table 1).

### Discussion

This first study aimed at eliciting an emotional response associated with the experience of guilt. We identified a pattern of facial movements produced more when experiencing guilt and reporting higher levels of guilt; people frowned (AU4, Brow Lowerer), stretched their lips (AU20, Lip Stretched) and touched their neck (Neck Touching), as well as looking towards the laptop from which they pulled out the USB stick (AUs52+62, Head Turn Right and Eyes Turn Right). Those specific behaviours were not associated with self-reported feelings of shame or pride. Previous research that identified behavioural displays associated with embarrassment (gaze down, controlled smiles, gaze shifts, and face touches [44]), shame (head and gaze down [43–45]) and pride (expanded posture, head tilted back, low-intensity non-Duchenne smile [76]) did not report AUs4+20, gaze right and Neck Touch as part of those displays. Therefore, these components seem specific to the experience of self-reported guilt that the participants reported in our study. This is the first study to identify a potential pattern of movements associated with felt guilt.

To determine whether these movements were also identified as guilt by observers when presented with contextual information, we conducted a second study. We asked new participants to examine the videos of participants from this first study during the induction of guilt (Fig 1:4) and rate them for emotion. We also asked these new participants to identify specific times when they thought they could see these specific emotions. This study aimed at identifying which specific facial movements were most closely associated with the perception of guilt.

## Study 2 –Judgement of guilt

### Methods

#### Participants

One hundred and fourteen new participants (hereafter referred to as “judges”) were recruited for this second study (82 females; *M*_*age*_ = 29.96, *SD* = 11.48; see SM3 for details). None of the participants that took part in Study 1 was recruited for Study 2. As in Study 1, judges were recruited based on an opportunistic sampling method and were all UK residents at the time of the experiment (but included both UK and non-UK nationals; see S1 Study of Table 1 for details). All of them received either course credit (if a student) or £5 for their time. The whole experiment lasted approximately 35 minutes. The project has been reviewed and approved by the Science Faculty Ethics Committee (SFEC) from the University of Portsmouth. Each participant signed an informed consent form granting authorisation for the use of the data for research purposes.

#### General procedure

At the start, participants were given general instructions regarding the experiment and written consent was obtained. Judges were originally told that this study had a different aim—to assess their abilities to detect facial expressions of emotion. We wanted to test whether people could see guilt on a face without actively probing guilt detection (i.e., asking only about guilt). Following this, the rest of the instructions and tasks were displayed on a computer through Qualtrics Survey Software [77]. This experiment consisted of two successive tasks. Firstly, judges were asked to complete a personality questionnaire, the Guilt and Shame Proneness Scale [GASP, 78; order of questions was randomised between subjects]. Secondly, judges were asked to watch 20 consecutive videos of faces, and make a series of ratings regarding the emotional state of the stimulus individual (see below for more detail). Finally, the judges were informed about the true aim of the experiment and debriefed. The experiment was presented on desktop computers in one of the laboratories available at the University. Judges sat in front of the computer, the screen situated approximately 60cm away from their faces (face stimuli visual angle: 10° x 14°). The Qualtrics survey was presented in full-screen mode; videos were uploaded on Youtube, on a private account, and presented on Qualtrics as an embedded file. Judges had the opportunity to watch each video as many times as they wished to, and they could view it full screen. They could slow down the video but not watch it frame by frame and could scroll through the video.

#### Stimuli

All experimental video stimuli were taken from Study 1 (participants experiencing the guilt induction task, in the guilt condition). Of the 64 participants allocated to the guilt condition in Study 1, we used 57 individuals (seven participants were omitted for spending 50% of the time or more out of sight). Control video stimuli were also taken from Study 1 (participants from the control condition). For this, 12 individuals were chosen randomly. All 57 guilt videos were clipped to 30–90 seconds, all 12 control videos were 7 seconds long on average, and audio was removed. The stimuli were generally centred in the video but participants in Study 1 were free to move their head and body (see S1 Video).

#### Guilt judgements

Each judge watched 20 videos in succession (30- to 90-second-long videos), 16 guilt videos, and 4 control videos, out of the 69 videos selected for this study. The videos presented were randomised for each participant. Before viewing the videos, judges were provided with the following contextual information—‘the individual in the video had just been told they had wiped some important information from a USB flash drive’. The same contextual information was provided for all videos, guilt and control. This allowed us to test how many emotional expressions participants perceived in a specific guilt context. As previous research emphasised the importance of context in understanding facial expressions [41,79,80], we included contextual information to collect accurate, genuine, ecologically valid judgements. If participants were relying solely on the written context, they would see guilt on every face. However, if they looked at the face and used facial expressions to identify the emotional states, they would be able to see other emotions as well as guilt. While watching each video or right after viewing, judges were required to indicate how they thought the individual was feeling overall, using a sliding-scale (from 0–100%) for the five following emotional states: “uncomfortable”, “embarrassed”, “guilty”, “surprised”, and “other” (see S3 Fig). Those judgements were collected for the entire video, as a measure of the different emotional states the individual in the video seemed to experience, providing the judged guilt variable used in further analysis. Those five emotional states were selected based on the results from Study 1 [AUs indicative of these emotions; 20,39].

In addition to the sliding-scale rating, judges were encouraged to report any instances of emotion, i.e. any moment within the video were the emotion occurred (hereafter, a pinpoint), allowing for their judgements to be localised to an exact time point. They could have reported that overall the individual in the video experienced 20% of discomfort; this allowed them to indicate when exactly in the video was the individual experiencing discomfort. They were encouraged to report times when the indicated emotion was the most clearly expressed on the face (i.e., apexes of emotional expressions). To do so, judges could stop the videos whenever they wanted, watch the video multiple times, and even slow down the videos. Judges could not report a specific frame in the video due to the format of the stimuli, but they could report specific time (min:sec). Judges could make multiple pinpoints for multiple emotions, and multiple pinpoints per emotion. For example, they could report that in a video, the individual appears 50% guilty at 15 and 25 seconds in the video; or a judge could provide us with the information that an individual in a given video appears 50% embarrassed and 10% surprised at 35 seconds in the video, and 30% guilty at 40 seconds in the video.

When looking at the raw data, 623 instances of guilt were identified by all judges. This gave us a gross overview of the pinpoints reported. Some of these instances might be the same pinpoint (or unique instance), as multiple judges might have reported the same specific time. Moreover, 1,077 instances of surprise were reported, as well as 825 instances of discomfort and 676 instances of embarrassment. Judges seemed able to conceptually differentiate between those four emotional states as very few overlaps were made between them (see the “*Descriptive analysis”* sub-section in Results for details on guilt pinpoints).

#### Compiling the dataset

*Guilt*. Before analysis, the judgement data collected was combined with the FACS data produced in Study 1. The judges in this study reported 403 unique instances of guilt across the guilt videos and 36 unique instances across control videos, as identified by time-specific pinpoints on the video. We allowed for 0.5 seconds (or 12 video frames) of error around pinpoints, providing us with one second of video data per pinpoint in which judged guilt could have occurred. These pinpoints were synchronised with the FACS coding of the videos, to match judged guilt with any possible facial movements. We created these windows as the actual pinpoints reported by the judges were lacking precision; when synchronising the pinpoints with the FACS coding, we reported pinpoints in the middle of the second identified. For instance, a pinpoint identified by judges at 5 sec would be reported in the FACS coding at 5 sec 500 msec. The 1-second window allowed us to capture the movements they perceived as reporting guilt. Moreover, as genuine expressions have been shown to have onsets ranging from 0.50 to 0.70 sec [81,82], creating 1-second window around the identified pinpoints allowed us to capture the facial movements identified as conveying guilt by the judges. Multiple guilt windows could thus be created for a given video. We were not interested in capturing the unfolding of the entire expressions associated with guilty feelings, from onset to offset; rather we wanted to explore facial movements people associate with guilt. Thus, we allowed judges to watch the entire video and decide when guilt was most present on the face. Finally, any video data that occurred outside of these pinpoints (i.e. any part of the video that was not judged as guilty by any judge) was removed, providing us with a reduced dataset containing only judged guilt video frames. The creation of the pinpoints and removing all frames occurring outside the pinpoints resulted in 8,934 video frames of FACS data (present/absence of AU/ADs) from the guilt videos and 850 video frames from the control videos. This step was conducted to focus our data more on facial movements the judges could be considering guilty, and to reduce noise in the dataset. All the selected frames were retained for further analysis. We used the same 15 AUs as identified in Study 1 to run the following analysis (see S1 Study of Table 2 for details).

#### Statistical analysis

*Guilt*. First, to examine the judges’ ability to accurately perceive guilt on a face, we ran a Pearson’s correlation between the self-reported feeling of guilt of each participant and the averaged judged guilt per participant (i.e., video).

*Analysis of pinpoints*. To test how the frames chosen by the judges as displaying guilt differed from those frames that were not judged to display guilt, we conducted analyses following the same method as described in Study 1. Here, we compared a) the action units in the pinpoints for the twelve videos from the control condition, which were rated with all other frames of the same videos, and b) the action units in the pinpoints for the 57 videos from the experimental condition with all other frames from the same videos. Again, we created control distributions based on bootstraps for the control data (in both cases, non-pinpointed frames of the control and experimental condition, respectively) and we tested whether the occurrence of action units in the test data differed. Again the randomisation was based on the level of individuals and we controlled for the PoO of participants chosen for the control distribution. We removed all frames that did not contain any action units from these analyses, as they would not be chosen by judges as displaying guilt.

*Judged guilt*. To test whether participants judged to display an overall higher level of guilt differed in their properties or facial activity from those that were not judged to display guilt, we fitted a linear mixed model [83] with Gaussian error structure. Analyses were conducted in R v.3.6.1 [84]. We focused on the presence of four action units (AU4, AU10, AU20, Neck Touch) that were consistently produced more often in the different guilt conditions (see Study 1), and tested whether increased production of these signals in a video also increased how guilty the participant looked. The average guilt rating of judges (range 9.5–54.4, mean 32.8) was set as the response variable, and followed a normal distribution. Guilt ratings were available for 69 videos. Given that the guilt ratings for each video were averaged across judges, there were no random effects. As predictor variables, we set the ratio of frames in each video that contained AU4, AU10, AU20, and neck touching; a variable indicating how many of these four action units were observed in a video; the condition (control, experiment); and PoO of the participant.

All continuous variables were z-standardized to facilitate interpretation [85]. We compared the full model against a null model only containing the PoO, the condition, and the self-reported guilt change, to test whether the facial activity influenced perceived guilt at all [86]. To establish the significance of each predictor variable, we tested the full model against a reduced model not containing the variable [87] using the ‘drop1’ function in R. We tested for collinearity using Variance Inflation Factors [88] with the ‘vif’ function in the ‘car’ package [89]; collinearity of test variables was not an issue (maximum VIF 1.84).

### Results

#### Descriptive statistics

Overall, judges attributed a higher level of guilt to participants in the guilt videos (*M* = 35.65, *SD* = 9.46) compared to participants in control videos (*M* = 19.16, *SD* = 7.47; t(19.246) = -6.61, p < 0.001). They also attributed higher level of surprise to the guilt videos (*M* = 42.46, *SD* = 16.18) compared to the control videos (*M* = 21.48, *SD* = 12.93; t(2.051) = -4.88, p < 0.001).

The judges reported 403 instances of guilt across the guilt videos, with an average of seven pinpoints per video, and 36 instances across the control videos. In 40 of those instances (10% of the total amount of guilt pinpoints identified across all videos), guilt was associated with one other emotion (guilt was associated with embarrassment in 45% of these 58 occurrences, with discomfort for 47.5% and surprise for 7%; see S1 Study of Table 2).

Judges made reliable ratings regarding the level of felt guilt: we found a positive correlation between the averaged judged guilt per individual and the individual self-reported guilt (r = 0.465, n = 69, p < 0.001).

#### Analysis of pinpoints

*Control videos*. For the frames that were identified by judges in the videos belonging to the control condition of the experiment, the pinpointed frames did only differ significantly from other frames by showing more activity in AU20 and in face touching (Table 4). They also showed less activity of AU10 and AU 61.

**Table 4:** Comparison of the dataset for the frames from control videos that were selected as displaying guilt by judges (observed frequency) with the dataset containing the remaining frames for the control videos (expected frequency), after controlling for differences in the reported PoOs of participants.

AU	Observed Frequency	Expected Frequency	z	p-value
1	0.41	0.53	-1.25	0.096
2	0.41	0.53	-1.25	0.096
4	0.05	0.02	1.86	0.033
5	0.05	0.04	1.14	0.122
7	0.34	0.44	-0.87	0.184
*10*	*0*.*03*	*0*.*22*	*-2*.*22*	*0*.*005*
12	0.35	0.36	-0.12	0.414
14	0.29	0.45	-1.51	0.059
17	0.10	0.26	-1.52	0.109
18	0.01	0.07	-1.80	0.035
**20**	**0.04**	**0.01**	**7.82**	**0.000**
24	0.05	0.07	-0.69	0.288
51	0.21	0.37	-1.83	0.037
52	0.19	0.38	-1.94	0.049
54	0.20	0.14	1.56	0.067
55	0.11	0.21	-1.57	0.068
56	0.35	0.22	1.82	0.044
57	0.20	0.18	0.34	0.342
59	0.01	0.04	-1.54	0.079
*61*	*0*.*16*	*0*.*26*	*-2*.*55*	*0*.*003*
62	0.12	0.18	-1.48	0.065
64	0.51	0.49	0.37	0.334
**FaceTouch**	**0.10**	**0.04**	**3.43**	**0.002**
NeckTouch	0.00	0.00	0.00	1.000

P-values denote the likelihood that the observed frequency of occurrence for an AU was more extreme than the predicted frequency. AU with significantly increased occurrence in bold, AU with significantly decreased occurrence in italics.

*Guilt videos*. For the pinpoints selected by judges in guilt videos, these frames differed substantially from other frames in the same videos (Table 5). They had increased activity for AU4, AU5, AU17, AU54, AU61, AU62, AU64, and for neck touching. They had decreased activity AU57 and AU59. These results mirror some of the signals of guilt in Study 1, further evidence that AU4, AU20, and self-directed behaviour (neck touching) showed differences in production and perception of guilt. Judges seemed to use increased eye movement (AU61, AU62, AU64) as a sign of guilt.

**Table 5:** Comparison of the dataset for the frames from guilt videos that were selected as displaying guilt by judges (observed frequency) with the dataset containing the remaining frames for the same guilt videos (expected frequency), after controlling for differences in the reported PoO of participants.

AU	Observed Frequency	Expected Frequency	z	p-value
1	0.56	0.52	1.11	0.142
2	0.53	0.51	0.53	0.287
**4**	**0.26**	**0.20**	**2.54**	**0.006**
**5**	**0.15**	**0.11**	**2.98**	**0.004**
7	0.29	0.26	0.59	0.276
10	0.33	0.27	2.00	0.017
12	0.16	0.15	0.42	0.355
14	0.15	0.17	-0.76	0.230
**17**	**0.20**	**0.13**	**5.25**	**0.000**
18	0.15	0.14	0.87	0.194
20	0.04	0.04	0.19	0.429
24	0.16	0.15	0.67	0.238
51	0.08	0.10	-1.22	0.101
52	0.31	0.30	0.36	0.370
**54**	**0.22**	**0.15**	**3.66**	**0.000**
55	0.20	0.21	-0.14	0.419
56	0.16	0.19	-0.97	0.193
*57*	*0*.*10*	*0*.*18*	*-3*.*94*	*0*.*000*
*59*	*0*.*02*	*0*.*03*	*-2*.*45*	*0*.*006*
**61**	**0.12**	**0.09**	**2.63**	**0.001**
**62**	**0.26**	**0.21**	**3.99**	**0.000**
**64**	**0.36**	**0.30**	**3.52**	**0.000**
FaceTouch	0.15	0.13	0.85	0.203
**NeckTouch**	**0.03**	**0.02**	**2.81**	**0.002**

P-values denote the likelihood that the observed frequency of occurrence for an AU was more extreme than the predicted frequency. AU with significantly increased occurrence in bold, AU with significantly decreased occurrence in italics.

#### Judged guilt

The full null model comparison revealed a significant impact of facial activity on judged guilt (X^2^ = 1774.8, df = 5, p < 0.001). Of the test predictors, the activity of AU4 in a video (X^2^ = 817.8, df = 1, p < 0.001) and the amount of neck touching (X^2^ = 234.4, df = 1, p = 0.041) both positively influenced perceived guilt, while AU10 and AU20 did not show any effect; neither did the overall number of the four action units present. There was no impact of the PoO of the participant, nor was there here an impact of the self-reported guilt. Videos from the guilt condition were rated as displaying higher guilt than videos from the control condition (X^2^ = 928.9, df = 1, p < 0.001).

### Discussion

This study aimed to identify which facial movements were perceived as guilt when guilt was induced in a laboratory experiment. We found that judges gave a higher rating of guilt in videos where people were seen frowning (AU4 Brow Lowerer) and touching their neck (Neck Touching). We used instances when judges reported seeing guilt to create 1s-window of interest and conduct our analysis only on those time windows of guilt. Doing this, we identified facial movements reliably associated with the perceived expression of guilty. Judges reported other emotions at the same time as guilt in only 14% of the guilt pinpoints. Moreover, pinpoints of guilt revealed specific facial movements that were not present in control videos. This made us fairly confident that the facial expressions identified were associated with the experience (perception) of guilt.

## General discussion

In two studies, we aimed to identify facial movements and behavioural displays associated with the experience of guilt in humans. In the first study, we examined the production of guilt using a novel induction technique. In the second study, we examined whether others perceived guilt from the face of those experiencing guilt. We used an extensive, bottom-up coding scheme to identify facial patterns associated with the experience (production and perception) of guilt as part of a dynamic sequence of behaviour, combined with a robust bootstrapping method to analyse our data.

We found a positive relationship between the level of self-reported guilt and the extent this individual was judged as feeling guilty by others. This supports the idea that guilt could have evolved as an observable phenomenon with a potential communicative social function. The patterns identified in this experiment showed some consistency between what people do when feeling guilty and what people see when identifying guilt. Our first study showed that guilt was associated with frowning, lip stretching and neck touching [AU4 Brow Lowerer, AU20 Lips Stretch; 59], as well as looking towards the right (AU52 Head Right, AU62 Eyes Right), which was probably an artefact of the position of the computer. Our second study showed that the identification of guilt in others was associated with frowning, eyes widening, and neck touching [AU4 Brow Lowerer, AU5 Upper Lid Raiser, AU10 Upper Lip Raiser; 59], as well as looking down and sideways (AU54 Head Down, AU61 Eyes Left, AU62 Eyes Right, AU64 Eyes Down), another potential artefact due to the experimental set-up. Thus, it seems that in this study, guilt was associated with a non-verbal pattern of frowning and neck touching.

Using a bottom-up methodology allowed us not only to approach our question without any a priori assumptions regarding the results, but it also increased the likelihood that the movements identified in our studies (AU4, AU20, and neck touch) are associated with the experience of guilt and no other secondary moral emotion. Indeed, the “guilt” pinpoints identified by the judges (Study 2) were mainly instances of identification of guilt alone, with only 14% of the total number of guilt pinpoints associated with more than one emotion (see S1 Study of Table 2). This allowed us to focus our analysis on facial movements associated with the experience of guilt only. Moreover, even though guilt is often mistaken for embarrassment or shame, the embarrassed display has been characterised by the joint production of gaze down, controlled smiles, head turns, gaze shifts, face touches [44], and the occasional blushing [90]; and the typical face of shame was described with head and gaze movements down [43–45]. None of the movements we found associated with the expression of guilt were associated with those of other negative self-conscious emotions. During the AU selection process, most facial movements associated with either embarrassment or shame were discarded from further analysis, with the only exception of face touching. Face touch can emphasise embarrassment displays, but it is not necessary for the identification of embarrassment [44]. A previous study suggested a link between blushing and admission of guilt [91]; combining FACS analysis with thermal imaging techniques might have revealed changes in facial temperature in guilty participants, which could be unconsciously used by observers in their judgments.

This bottom-up methodology also diverges from previous research examining the facial display of guilt, which is why we may have found a more concrete candidate for the display of guilt. One notable previous study used a literature-based conceptualisation of the experience of guilt to present three candidates’ displays to their participants [8]. In that study, using a top-down approach, the participants were presented with displays selected based on previous literature, which associated the experience of guilt with the experience of self-contempt, sympathy, and pain. The authors tested whether their conceptualisation of guilt accurately described a facial display associated with the experience of the emotion. The results were not conclusive as the candidates’ displays were more often associated with emotions other than guilt [8]. A more recent study associated the experience of guilty feeling with increased skin conductance and gaze avoidance [92]. We did not find gaze avoidance (i.e. actively avoiding to look in another person’s direction) to be part of the facial signal of guilt, even though participants in the guilt condition looked down and around more than participants in the control condition. Yet, this could be due to our experimental design: participants in the guilt condition might have been looking down at the laptop more than people in the control condition. It is thus unclear in our design whether guilty participants avoided eye-contact or focused on an object associated to their wrongdoing (the laptop could be incriminated for the deletion of data on the USB stick, removing the fault from them).

Both the production and perception of guilt was associated with self-directed behaviour (i.e., scratching, neck or face touching), which are often classified as displacement behaviours, and are defined as a group of behaviours that appear irrelevant to the situation in which they are displayed, but can gain communicative value over time [61]. The production of such behaviours has been shown to increase in stressful, negative, situations [93,94]. Self-directed behaviours may be used when individuals try to distance and protect themselves from an unpleasant situation, acting as a short-term diversion of attention, which could, in turn, reduce the negative feeling associated to the situation at hand [93,95,96]. Self-directed behaviour could thus help regulate the level of stress associated with emotionally challenging situations [94], such as the guilt induction experienced by our participants in Study 1. Indeed, some studies have shown that self-directed behaviours are common in situations such as embarrassment [44], discomfort [20], and anxiety and guilt [97], which focussed on hand movements and found a correlation between the production of self-directed behaviours (i.e., scratching) and anxiety and guilt feelings. In our study, we found that the experience of guilt was associated with self-directed behaviours (neck touching), which appears to be in line with previous research. However, the production of self-directed behaviours could be due to the experimental design: participants were seated at a table, in front of a computer. However, the setup is unlikely to have elicited those movements, as participants in the control condition, also seated at a computer, did not display as many self-directed behaviours.

More recent conceptualisations of emotional experiences [27,28,35–37] argue for a less universal and omnipotent link between the experience of an emotion and behavioural outcomes. In an emotional context, multiple systems will be triggered (e.g., cognitive processes, physiological systems, motor expressions; [35]), leading to multiple behavioural outcomes (e.g. facial signals), one of which might be used by observers when responding to the situation [35]. As such, an individual feeling guilty might produce multiple facial signals, one of which will be more strongly associated with the subjective, constructed, feeling of guilt (e.g., frown, lips stretch and neck touching); an observer might perceive those facial signals and rely mainly on specific ones to interpret the emotional state of the guilty individual (e.g., frown and neck touching).

It is important to remain cautious in the interpretation of our data. We need to acknowledge that if neck touching was present more in association with feelings of guilt, only 12.5%of the individuals displayed neck touching. Self-directed behaviour, however, were displayed in over 64% of the individuals during the guilt induction. Even though few participants displayed neck touching, our results showed it is a significant signal of guilt. We need to consider the possibility that by reducing our dataset to 1-second windows, we could have excluded non-verbal signals important for the onset of the experience of guilt. By focussing on the apexes of the expressions, we might have lost secondary signals contributing to the reliable identification of guilty signals. Our results provide preliminary information regarding the non-verbal signals exhibited more in association with guilty feelings. A follow-up study, using a reduced ethogram focussing on the movements identified here could allow to reach a better agreement score between coders and thus increase the K’s alpha and the validity of our results [67,68]. We also need to consider the fact that providing contextual information might have influenced the judges in their decisions. To assess the impact of context, we conducted a follow-up study comparing the judgements made with and without contextual information provided [98]. Our judgement study also presents some linguistic limitations. Even if there are differences in the appraisal and behavioural outcomes between shame and guilt, it has been previously shown that English speaker use “guilt” and “shame” interchangeably [99]. To overcome this conceptual barrier, we conducted another judgement study, without providing contextual information [98,100]. We hope to gauge how the expression of guilt is perceived when no verbal/written content needs to be understood first. Moreover, to compare various judgement methodologies [emotion words vs action tendencies vs dimensions; 101], we conducted another follow-up study to help us have a better understanding of how people conceptualise the facial expression produced when experiencing guilt, using different types of words and classification methodologies [forced choice vs free labelling vs dimensions; 100]. This way, we hoped to introduce more variability in the emotional judgements, looking at patterns of mislabelling of guilty displays.

These are the first studies to look at the genuine expression of guilt and the perception of secondary emotion using spontaneous dynamic stimuli. Judges had to rely on genuine, dynamically presented facial expressions to recognise and rate emotions. They were exploratory studies, using simple analysis and focussing on the behavioural signals associated with a guilt-inducing situation. We have however collected more extensive data; now that we identified a facial signal associated with the experience of guilt, more in-depth analysis (such as a lens modelling [35]) would be an interesting step to further break down the mechanisms associated with guilt.

Our experiments support a drive towards a new scientific culture, studying facial expressions using novel approaches removed from the dichotomous debate about nature vs nurture [73,102]. Previous research extensively looked at the behavioural consequences of guilty feelings: it can promote directed action towards those who have been wronged [4], it can reduce prejudice behaviours [13] and increase generosity [6]. We focussed on the first reactions people have when realising they did something wrong and the guilty feelings emerge; we were able to identify reliable candidates characterising the experience of self-reported guilt. Building on this, we conducted a study to investigate guilty people’s propensity to repair the relationship, as well as the impact of a facial expression on the person wronged, i.e. the victim, reaction [103]. Together, our results suggest that guilt is expressed on the face and communicates the experience of guilt to others through a signal.

## Supporting information

S1 Fig Post-induction affect change. The variations in self-reported affect (guilt, shame, distress, and pride) are presented for each participant (grey dots/lines) before and after induction (see Fig 1 for details). The central tendencies presented in S1 Study of Table 3 are displayed here by the thick line.
(TIF)

S2 Fig Representation of the temporal production of facial movements. The number of participants produced each AU through time is presented on this heat map. Cells in white indicate that the AU was produced equally in the guilt condition and the control condition at a given time; cells in red indicate the AU was produced more by participants in the guilt condition; cells in blue indicate the AU was more produced by participants in the control condition. Time is presented in seconds. Gradients of red and blue represent the difference between the proportion of participants displaying AU in guilt condition and the proportion of participants displaying AU in control condition; the dark the colour, the greater the difference (*no statistical analysis conducted here*). The patterns (dots and lines) were added to help increase the readability of the figure: cells with dots mean the AU was more produced in the guilt condition at this time and cells with lines mean the AU was more produced in the control condition (how much more produced is given by the shape of the colour).
(TIF)

S3 Fig Judgement ratings. A representation of the different judgements made by judges: a. ruler ratings on 5 emotions; b. reporting pinpoints section; c. overall confidence regarding judgement.
(TIF)

S1 Video Video taken during the guilt induction task presenting the succession of AUs produced by a participant. (AVI)

S1 Data (XLSX)

S2 Data (XLSX)

S3 Data (XLSX)

S4 Data (XLSX)

S5 Data (XLSX)

S6 Data (XLSX)

S7 Data (XLSX)

S8 Data (XLSX)

S9 Data (XLSX)

S10 Data (XLSX)

S11 Data (XLSX)

S1 File (R)

S2 File (R)

S3 File (R)

S1 Study (DOCX)

## References

[1] Tomasello M (2008) Origins of human communication. Cambridge, MA: MIT Press.

[2] Haidt J (2003) The moral emotions In: Davidson RJ, Schere KR, Goldsmith HH, editors. Handbook of affective sciences. Oxford University Press pp. 852–870.

[3] Carroll J (1985) Guilt: the grey eminence behind character, history, and culture: Routledge & Kegan Paul Books.

[4] Cryder CE, Springer S, Morewedge CK (2012) Guilty Feelings, Targeted Actions. Personality and Social Psychology Bulletin 38: 607–618. 10.1177/0146167211435796 22337764 PMC4886498

[5] De Hooge IE, Zeelenberg M, Breugelmans SM (2007) Moral sentiments and cooperation: Differential influences of shame and guilt. Cognition and Emotion 21: 1025–1042.

[6] Ketelaar T, Au WT (2003) The effects of feelings of guilt on the behaviour of uncooperative individuals in repeated social bargaining games: An affect-as-information interpretation of the role of emotion in social interaction. Cognition & Emotion 17: 429–453. 29715746 10.1080/02699930143000662

[7] Weisman MR (2014) Showing remorse: Law and the social control of emotion: Ashgate Publishing, Ltd.

[8] Keltner D, Buswell BN (1996) Evidence for the distinctness of embarrassment, shame, and guilt: A study of recalled antecedents and facial expressions of emotion. Cognition & Emotion 10: 155–171.

[9] Keltner D, Gross JJ (1999) Functional accounts of emotions. Cognition & Emotion 13: 467–480.

[10] Muris P, Meesters C (2014) Small or big in the eyes of the other: on the developmental psychopathology of self-conscious emotions as shame, guilt, and pride. Clinical child and family psychology review 17: 19–40. 10.1007/s10567-013-0137-z 23712881

[11] Vaish A (2018) The prosocial functions of early social emotions: the case of guilt. Current opinion in psychology 20: 25–29. 10.1016/j.copsyc.2017.08.008 28830002

[12] Zemack-Rugar Y, Bettman JR, Fitzsimons GJ (2007) The effects of nonconsciously priming emotion concepts on behavior. Journal of personality and social psychology 93: 927 10.1037/0022-3514.93.6.927 18072846

[13] Amodio DM, Devine PG, Harmon-Jones E (2007) A dynamic model of guilt implications for motivation and self-regulation in the context of prejudice. Psychological Science 18: 524–530. 10.1111/j.1467-9280.2007.01933.x 17576266

[14] Ohbuchi K-i, Kameda M, Agarie N (1989) Apology as aggression control: its role in mediating appraisal of and response to harm. Journal of personality and social psychology 56: 219 10.1037//0022-3514.56.2.219 2926625

[15] O'Malley MN, Greenberg J (1983) Sex differences in restoring justice: The down payment effect. Journal of Research in personality 17: 174–185.

[16] Nelissen R, Zeelenberg M (2009) When guilt evokes self-punishment: evidence for the existence of a Dobby Effect. Emotion 9: 118 10.1037/a0014540 19186924

[17] Baumeister RF, Stillwell AM, Heatherton TF (1994) Guilt: an interpersonal approach. Psychological bulletin 115: 243 10.1037/0033-2909.115.2.243 8165271

[18] Crivelli C, Fridlund AJ (2019) Inside-Out: From Basic Emotions Theory to the Behavioral Ecology View. Journal of Nonverbal Behavior: 1–34. 10.1007/s10919-018-0289-0 31148883 PMC6514200

[19] Ekman P, Cordaro D (2011) What is meant by calling emotions basic. Emotion review 3: 364–370.

[20] Ekman P, Friesen WV (1969) The repertoire of non-verbal behavior: Categories, origins, usage and coding. Semiotica 1: 49–98.

[21] Brown DE (1991) Human universals: McGraw-Hill New York.

[22] Ekman P, Keltner D (1970) Universal facial expressions of emotion. California Mental Health Research Digest 8: 151–158.

[23] Bedford OA, Hwang KK (2003) Guilt and Shame in Chinese Culture: A Cross‐cultural Framework from the Perspective of Morality and Identity. Journal for the Theory of Social Behaviour 33: 127–144.

[24] Matsumoto D, Yoo SH, Hirayama S, Petrova G (2005) Development and validation of a measure of display rule knowledge: the display rule assessment inventory. Emotion 5: 23 10.1037/1528-3542.5.1.23 15755217

[25] Tangney JP (1999) The self-conscious emotions: Shame, guilt, embarrassment and pride.

[26] Izard CE (1994) Innate and universal facial expressions: evidence from developmental and cross-cultural research. 10.1037/0033-2909.115.2.288 8165273

[27] Fridlund AJ (1994) Human facial expression—An evolutionary view. London: Academic Press.

[28] Fridlund AJ (2017) The behavioral ecology view of facial displays: 25 years later In: Russell J-MF-DJA, editor. The science of facial expression: Oxford University Press pp. 77–92.

[29] Ekman P, Friesen WV (1971) Constants across cultures in the face and emotion. Journal of personality and social psychology 17: 124 10.1037/h0030377 5542557

[30] Waller BM, Whitehouse J, Micheletta J (2016) Macaques can predict social outcomes from facial expressions. Animal cognition 19: 1031–1036. 10.1007/s10071-016-0992-3 27155662 PMC4967087

[31] Krebs JR, Davies N (1993) An introduction to behavioural ecology: Blackwell Scientific Publications.

[32] Krebs JR, Dawkins R (1978) Animal signals: information or manipulation In: Krebs JR, Dawkins R, editors. Behavioural ecology: An evolutionary approach: Blackwell Scientifc Publications pp. 282–309.

[33] Dezecache G, Mercier H, Scott-Phillips TC (2013) An evolutionary approach to emotional communication. Journal of Pragmatics 59: 221–233.

[34] Bradbury JW, Vehrencamp SL (1998) Principles of animal communication. 10.1006/anbe.1999.1330 10675247

[35] Scherer KR, Mortillaro M, Mehu M (2013) Understanding the mechanisms underlying the production of facial expression of emotion: A componential perspective. Emotion Review 5: 47–53.

[36] Barrett LF (2017) The theory of constructed emotion: an active inference account of interoception and categorization. Social cognitive and affective neuroscience 12: 1–23. 10.1093/scan/nsw154 27798257 PMC5390700

[37] Barrett LF (2017) How emotions are made: The secret life of the brain: Houghton Mifflin Harcourt.

[38] Barrett LF, Mesquita B, Gendron M (2011) Context in emotion perception. Current Directions in Psychological Science 20: 286–290.

[39] Ekman P. Universals and cultural differences in facial expressions of emotion; 1971 University of Nebraska Press.

[40] Teroni F, Deonna JA (2008) Differentiating shame from guilt. Consciousness and cognition 17: 725–740. 10.1016/j.concog.2008.02.002 18445530

[41] Aviezer H, Hassin R, Bentin S, Trope Y (2008) Putting facial expressions back in context. First impressions: 255–286.

[42] Hess U, Blaison C, Kafetsios K (2016) Judging facial emotion expressions in context: The influence of culture and self-construal orientation. Journal of Nonverbal Behavior 40: 55–64.

[43] Izard CE (1977) Human emotions. New-York: Plenum Press.

[44] Keltner D (1995) Signs of appeasement: Evidence for the distinct displays of embarrassment, amusement, and shame. Journal of personality and social psychology 68: 441.

[45] Lewis M, Alessandri SM, Sullivan MW (1992) Differences in shame and pride as a function of children's gender and task difficulty. Child development 63: 630–638. 1600827

[46] Keltner D, Anderson C (2000) Saving face for Darwin: The functions and uses of embarrassment. Current directions in psychological science 9: 187–192.

[47] Higgins ET (1987) Self-discrepancy: a theory relating self and affect. Psychological review 94: 319 3615707

[48] Eisenberg N, Fabes RA, Miller PA, Fultz J, Shell R, et al (1989) Relation of sympathy and personal distress to prosocial behavior: a multimethod study. Journal of personality and social psychology 57: 55 10.1037//0022-3514.57.1.55 2754604

[49] Emde RN, Johnson WF, Easterbrooks MA (1987) The do's and don'ts of early moral development: Psychoanalytic tradition and current research. The emergence of morality in young children: 245–276.

[50] Henrich J, Heine SJ, Norenzayan A (2010) The weirdest people in the world? Behavioral and brain sciences 33: 61–83. 10.1017/S0140525X0999152X 20550733

[51] Galati D, Sini B, Schmidt S, Tinti C (2003) Spontaneous facial expressions in congenitally blind and sighted children aged 8–11. Journal of Visual Impairment and Blindness 97: 418–428.

[52] Schmidt KL, Cohn JF, Tian Y (2003) Signal characteristics of spontaneous facial expressions: Automatic movement in solitary and social smiles. Biological psychology 65: 49–66. 10.1016/s0301-0511(03)00098-x 14638288 PMC2839541

[53] Mathôt S, Schreij D, Theeuwes J (2012) OpenSesame: An open-source, graphical experiment builder for the social sciences. Behavior research methods 44: 314–324. 10.3758/s13428-011-0168-7 22083660 PMC3356517

[54] Gosling SD, Rentfrow PJ, Swann WB (2003) A very brief measure of the Big-Five personality domains. Journal of Research in Personality 37: 504–528.

[55] Jonason PK, Webster GD (2010) The Dirty Dozen: A Concise Measure of the Dark Triad. Psychological Assessment 22: 420–432. 10.1037/a0019265 20528068

[56] Watson D, Clark LA, Tellegen A (1988) Development and validation of brief measures of positive and negative affect: the PANAS scales. Journal of personality and social psychology 54: 1063 10.1037//0022-3514.54.6.1063 3397865

[57] Rebega OL, Apostol L, Benga O, Miclea M (2013) Inducing Guilt: A Literature Review. Procedia-Social and Behavioral Sciences 78: 536–540.

[58] De Hooge IE, Nelissen R, Breugelmans SM, Zeelenberg M (2011) What is moral about guilt? Acting “prosocially” at the disadvantage of others. Journal of personality and social psychology 100: 462 10.1037/a0021459 21244173

[59] Ekman P, Friesen WV (1978) Facial action coding system. Palo Alto: Consulting Psychologists Press.

[60] Ekman P, Friesen WV, Hager JC (2002) Facial action coding system—investigator’s guide. Research Nexus, Salt Lake City.

[61] Troisi A (2002) Displacement activities as a behavioral measure of stress in nonhuman primates and human subjects. Stress 5: 47–54. 10.1080/102538902900012378 12171766

[62] Martin P, Bateson P (1993) Measuring behaviour: an introductory guide: Cambridge University Press.

[63] Mangold P (1998) Interact [computer software]. Arnstorf, Germany: Mangold International.

[64] Krippendorff K (1970) Bivariate agreement coefficients for reliability of data. Sociological methodology 2: 139–150.

[65] Hayes AF, Krippendorff K (2007) Answering the call for a standard reliability measure for coding data. Communication methods and measures 1: 77–89.

[66] IBM C (2016) SPSS for Windows, version 24. IBM Corp Armonk (NY).

[67] Krippendorff K (2004) Reliability in content analysis: Some common misconceptions and recommendations. Human communication research 30: 411–433.

[68] Krippendorff K (2018) Content analysis: An introduction to its methodology: Sage publications.

[69] Snijders TA, Borgatti SP (1999) Non-parametric standard errors and tests for network statistics. Connections 22: 161–170.

[70] Cousins SD (1989) Culture and self-perception in Japan and the United States. Journal of Personality and Social Psychology 56: 124.

[71] De Leersnyder J, Mesquita B (2015) How salient cultural concerns shape emotions: A behavioral coding study on biculturals’ emotional frame switching.

[72] Jack RE, Caldara R, Schyns PG (2012) Internal representations reveal cultural diversity in expectations of facial expressions of emotion. Journal of Experimental Psychology: General 141: 19. 21517206 10.1037/a0023463

[73] Crivelli C, Russell JA, Jarillo S, Fernández-Dols J-M (2016) The fear gasping face as a threat display in a Melanesian society. Proceedings of the National Academy of Sciences 113: 12403–12407. 10.1073/pnas.1611622113 PMC5098662 27791137

[74] Crivelli C, Russell JA, Jarillo S, Fernández-Dols J-M (2017) Recognizing spontaneous facial expressions of emotion in a small-scale society of Papua New Guinea. Emotion 17: 337 10.1037/emo0000236 27736108

[75] Benjamini Y, Yekutieli D (2001) The control of the false discovery rate in multiple testing under dependency. The annals of statistics 29: 1165–1188.

[76] Tracy JL, Robins RW (2008) The nonverbal expression of pride: evidence for cross-cultural recognition. Journal of personality and social psychology 94: 516 10.1037/0022-3514.94.3.516 18284295

[77] Qualtrics s (2012) Qualtrics. Available from http://qualtrics.com.

[78] Cohen TR, Wolf ST, Panter AT, Insko CA (2011) Introducing the GASP scale: a new measure of guilt and shame proneness. Journal of personality and social psychology 100: 947 10.1037/a0022641 21517196

[79] Fernandez‐Dols JM, Sierra B, Ruiz‐Belda MA (1993) On the clarity of expressive and contextual information in the recognition of emotions: A methodological critique. European Journal of Social Psychology 23: 195–202.

[80] Hess U, Banse R, Kappas A (1995) The intensity of facial expression is determined by underlying affective state and social situation. Journal of personality and social psychology 69: 280.

[81] Cohn JF, Schmidt K (2003) The timing of facial motion in posed and spontaneous smiles Active Media Technology: World Scientific pp. 57–69.

[82] Schmidt KL, Bhattacharya S, Denlinger R (2009) Comparison of deliberate and spontaneous facial movement in smiles and eyebrow raises. Journal of nonverbal behavior 33: 35–45. 10.1007/s10919-008-0058-6 20333273 PMC2843933

[83] Baayen R (2008) A practical introduction to statistics using R. Analyzing Linguistic Data. Cambridge University Press.

[84] Team RD (2016) R: A Language and Environment for Statistical Computing. R Found Stat Comput.

[85] Schielzeth H (2010) Simple means to improve the interpretability of regression coefficients. Methods in Ecology and Evolution 1: 103–113.

[86] Schielzeth H, Forstmeier W (2008) Conclusions beyond support: overconfident estimates in mixed models. Behavioral Ecology 20: 416–420. 10.1093/beheco/arn145 19461866 PMC2657178

[87] Dobson AJ, Barnett AG (2008) An introduction to generalized linear models: Chapman and Hall/CRC.

[88] Field A, Miles J, Field Z (2012) Discovering statistics using R: Sage publications.

[89] Fox J, Weisberg S, Adler D, Bates D, Baud-Bovy G, et al (2012) Package ‘car’. Vienna: R Foundation for Statistical Computing.

[90] de Jong PJ, Dijk C (2013) Social effects of facial blushing: influence of context and actor versus observer perspective. Social and Personality Psychology Compass 7: 13–26.

[91] De Jong PJ, Peters ML, De Cremer D (2003) Blushing may signify guilt: Revealing effects of blushing in ambiguous social situations. Motivation and emotion 27: 225–249.

[92] Yu H, Duan Y, Zhou X (2017) Guilt in the eyes: Eye movement and physiological evidence for guilt-induced social avoidance. Journal of Experimental Social Psychology 71: 128–137.

[93] Mohiyeddini C, Bauer S, Semple S (2013) Displacement behaviour is associated with reduced stress levels among men but not women. PloS one 8: e56355 10.1371/journal.pone.0056355 23457555 PMC3573003

[94] Mohiyeddini C, Semple S (2013) Displacement behaviour regulates the experience of stress in men. Stress 16: 163–171. 10.3109/10253890.2012.707709 23017012

[95] Chance MR. An interpretation of some agonistic postures; the role of “cut-off” acts and postures; 1962 Academic Press London pp. 71–89.

[96] Sgoifo A, Braglia F, Costoli T, Musso E, Meerlo P, et al (2003) Cardiac autonomic reactivity and salivary cortisol in men and women exposed to social stressors: relationship with individual ethological profile. Neuroscience & Biobehavioral Reviews 27: 179–188. 10.1016/s0149-7634(03)00019-8 12732233

[97] Ekman P, Friesen WV (1972) Hand movements. Journal of communication 22: 353–374.

[98] Julle-Danière E, Whitehouse J, Harris C, Chung M, Vrij A, et al. (in prep) Guilt outside of context.

[99] Fessler D (2004) Shame in two cultures: Implications for evolutionary approaches. Journal of Cognition and Culture 4: 207–262.

[100] Julle-Danière E (2019) The expression, experience, and social consequences of guilt: A cross-cultural study: University of Portsmouth.

[101] Julle-Danière E, Whitehouse J, Vrij A, Gustafsson E, Waller BM (under review) Are there non-verbal signals of guilt? PLOS One. 10.1371/journal.pone.0231756 PMC7182233 32330158

[102] Jack RE, Sun W, Delis I, Garrod OG, Schyns PG (2016) Four not six: Revealing culturally common facial expressions of emotion. Journal of Experimental Psychology: General 145: 708. 27077757 10.1037/xge0000162

[103] Julle-Danière E, Whitehouse J, Vrij A, Gustafsson E, Waller BM (in prep) The social outcomes of experiencing and seeing guilt.

