# Supplementary figures and images for: Are there non-verbal signals of guilt?
Source: PLoS One. 2020 Apr 24;15(4):e0231756. doi: 10.1371/journal.pone.0231756 (PMC7182233; doi:10.1371/journal.pone.0231756)

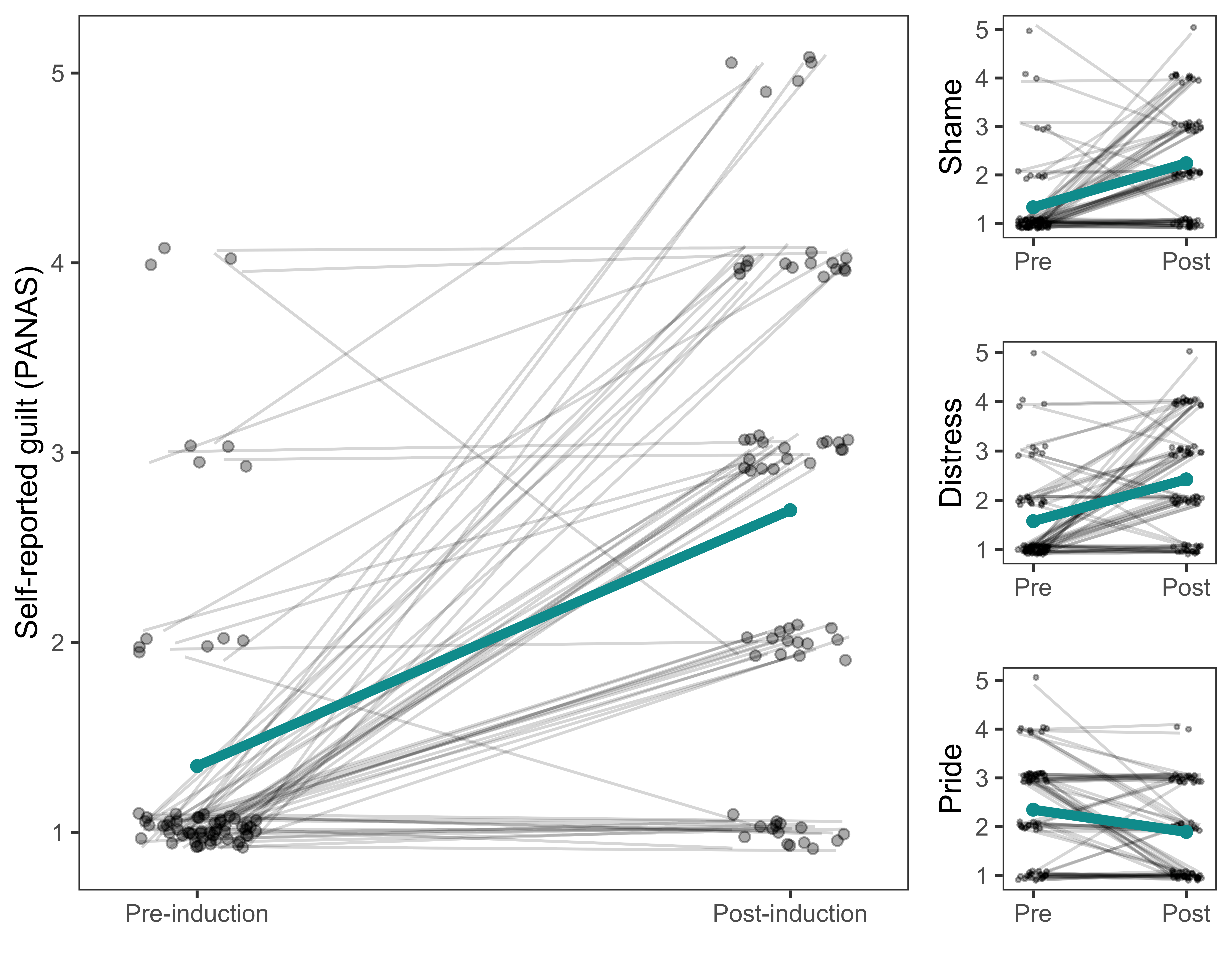

Supplement: S1 Fig — The variations in self-reported affect (guilt, shame, distress, and pride) are presented for each participant (grey dots/lines) before and after induction (see Fig 1 for details). The central tendencies presented in S1 Study of Table 3 are displayed here by the thick line. (TIF) [file pone.0231756.s001.tif]

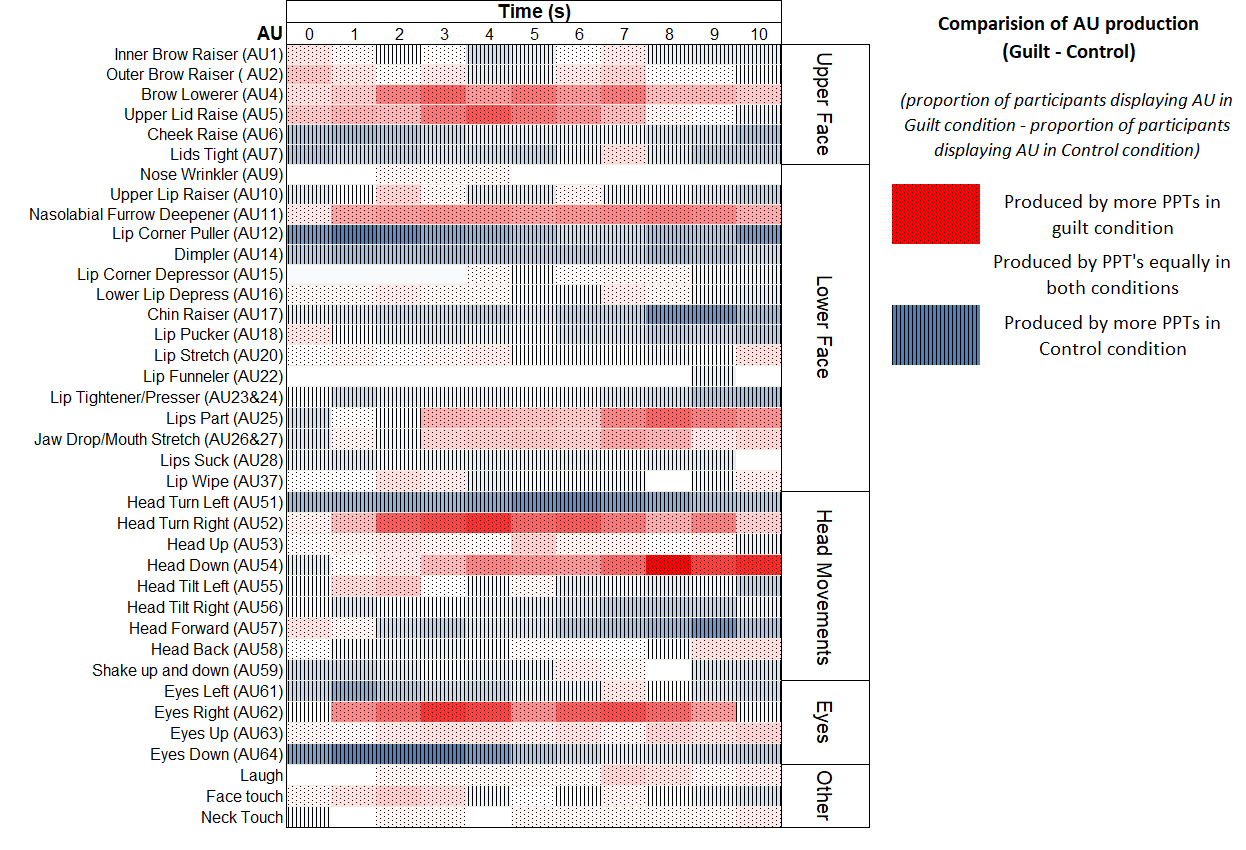

Supplement: S2 Fig — The number of participants produced each AU through time is presented on this heat map. Cells in white indicate that the AU was produced equally in the guilt condition and the control condition at a given time; cells in red indicate the AU was produced more by participants in the guilt condition; cells in blue indicate the AU was more produced by participants in the control condition. Time is presented in seconds. Gradients of red and blue represent the difference between the proportion of participants displaying AU in guilt condition and the proportion of participants displaying AU in control condition; the dark the colour, the greater the difference (no statistical analysis conducted here). The patterns (dots and lines) were added to help increase the readability of the figure: cells with dots mean the AU was more produced in the guilt condition at this time and cells with lines mean the AU was more produced in the control condition (how much more produced is given by the shape of the colour). (TIF) [file pone.0231756.s002.tif]

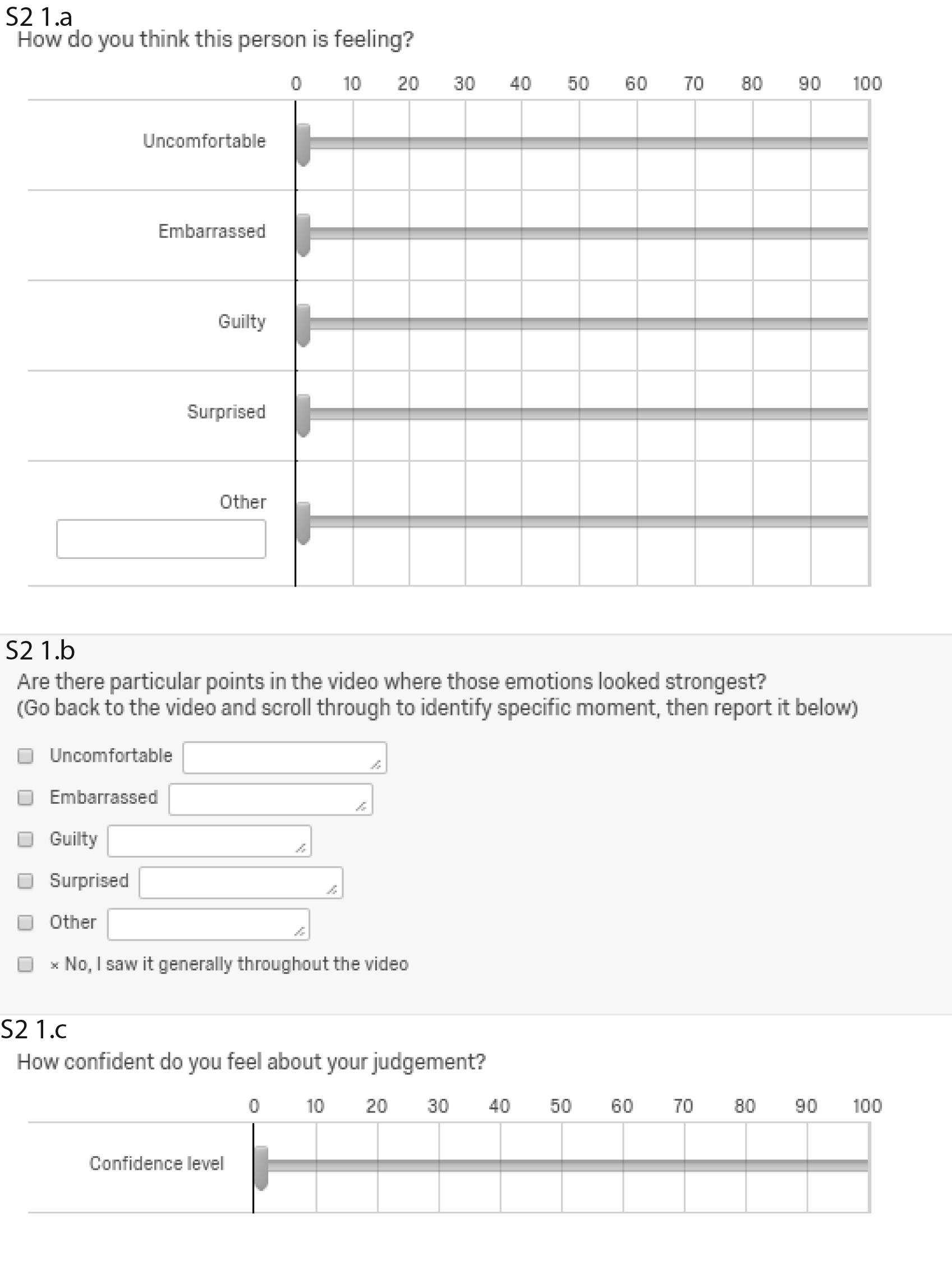

Supplement: S3 Fig — A representation of the different judgements made by judges: a. ruler ratings on 5 emotions; b. reporting pinpoints section; c. overall confidence regarding judgement. (TIF) [file pone.0231756.s003.tif]
