# Supplementary material for: Are there non-verbal signals of guilt?
Source: PLoS One. 2020 Apr 24;15(4):e0231756. doi: 10.1371/journal.pone.0231756 (PMC7182233; doi:10.1371/journal.pone.0231756)
Supplement: S1 Study — (DOCX) [file pone.0231756.s019.docx]

### Supporting information 1 - Study 1


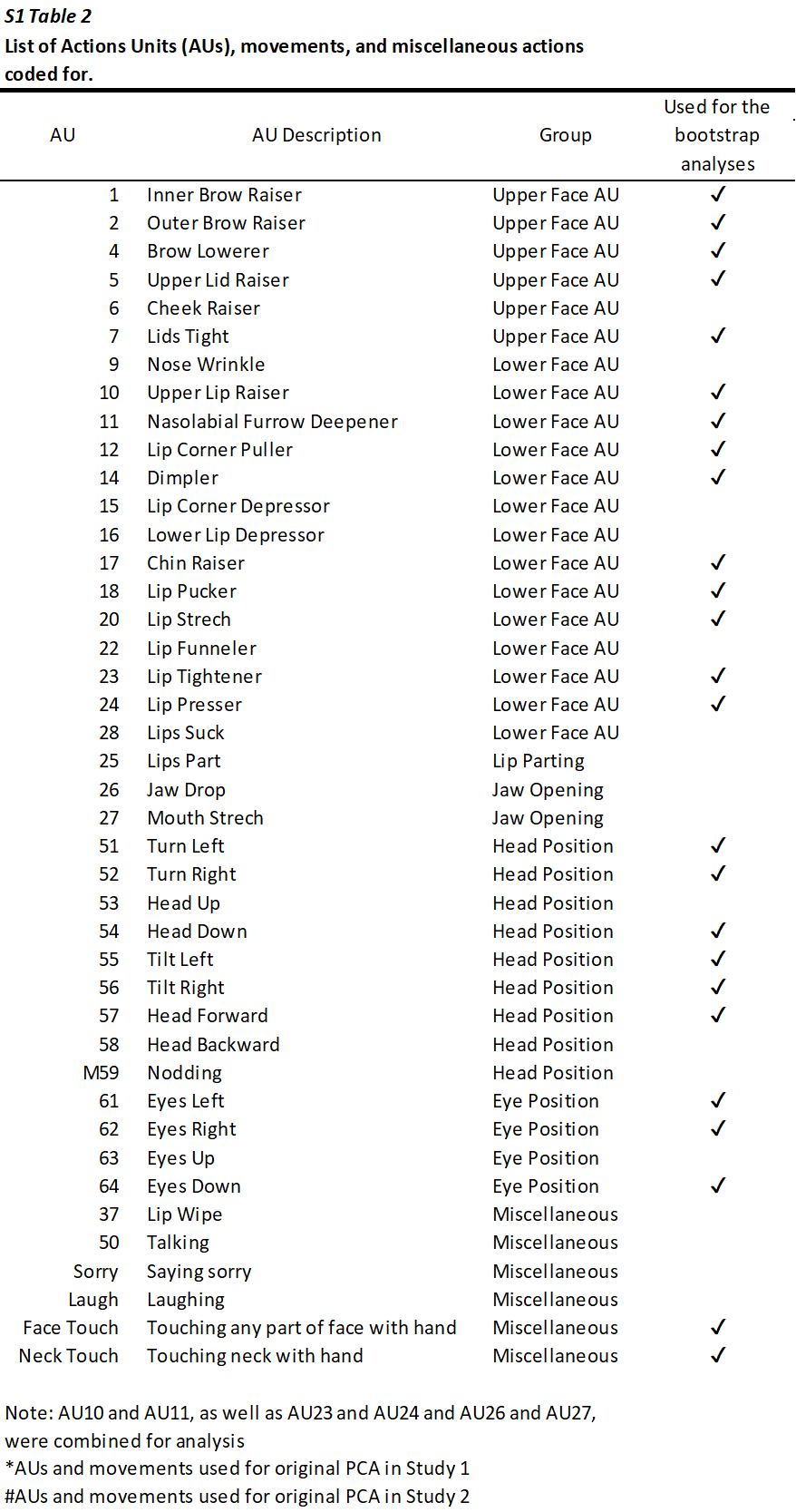


Personality data

Overall, participants from different cultural groups did not differ in agreeableness, emotional stability, extraversion, openness, machiavelism, psychopathy, or narcissism (p > 0.05). None of the personality traits correlated with the expressivity (p > 0.05), which means that any difference between groups is due to a cultural impact only. Machiavelism was positively correlated with the self-reported guilt (ß = 0.0674; SE = 0.0241; p < 0.001) and narcissism was negatively correlated with self-reported guilt (ß = -0.185; SE = 0.0907; p = 0.0439).

Results

Guilt induction (see S1 Table 3)

**S1 Table 3**

Self-reported emotions before and after guilt induction

### Supporting information 2 - Study 2
